# Leukocyte cell-derived chemotaxin 2 regulates epithelial-mesenchymal transition and cancer stemness in hepatocellular carcinoma

**DOI:** 10.1016/j.jbc.2022.102442

**Published:** 2022-08-31

**Authors:** Tian-Huei Chu, Chou-Yuan Ko, Po-Han Tai, Yi-Chen Chang, Chao-Cheng Huang, Tung-Yang Wu, Hoi-Hung Chan, Ping-Hsuan Wu, Chien-Hui Weng, Yu-Wei Lin, Mei-Lang Kung, Cheng-Chieh Fang, Jian-Ching Wu, Zhi-Hong Wen, Yung-Kuo Lee, Tsung-Hui Hu, Ming-Hong Tai

**Affiliations:** 1 Medical Laboratory, Medical Education and Research Center, Kaohsiung Armed Forces General Hospital, Kaohsiung, Taiwan; 2 Division of Gastroenterology and Hepatology, Department of Internal Medicine, Kaohsiung Armed Forces General Hospital, Kaohsiung, Taiwan; 3 Institute of Medical Science and Technology, National Sun Yat-sen University, Kaohsiung, Taiwan; 4 Institute of Biomedical Sciences, National Sun Yat-sen University, Kaohsiung, Taiwan; 5 Doctoral Degree Program in Marine Biotechnology, National Sun Yat-sen University and Academia Sinica, Kaohsiung, Taiwan; 6 Department of Pathology, Kaohsiung Chang Gung Memorial Hospital and Chang Gung University College of Medicine, Kaohsiung, Taiwan; 7 Department of Chest Medicine, Kaohsiung Armed Forces General Hospital, Kaohsiung, Taiwan; 8 Division of Gastroenterology, Department of Medicine, Conde S. Januário Hospital, Macau, China; 9 Department of Biological Sciences, National Sun Yat-sen University, Kaohsiung, Taiwan; 10 Department of Radiation Oncology, Kaohsiung Veterans General Hospital, Kaohsiung, Taiwan; 11 Department of Medical Education and Research, Kaohsiung Veterans General Hospital, Kaohsiung, Taiwan; 12 Center for Neuroscience, National Sun Yat-sen University, Kaohsiung, Taiwan; 13 LabTurbo Biotech Corporation, Taipei, Taiwan; 14 Department of Marine Biotechnology and Resources, Asia-Pacific Ocean Research Center, National Sun Yat-sen University, Kaohsiung, Taiwan; 15 Division of Hepato-Gastroenterology, Department of Internal Medicine, Kaohsiung Chang Gung Memorial Hospital, Chang Gung University College of Medicine, Kaohsiung, Taiwan

**Keywords:** hepatocellular carcinoma, leukocyte cell-derived chemotaxin 2, cancer stem cells, epithelial-mesenchymal transition, prognostic biomarker, Ad, adenovirus, cDNA, complementary DNA, CSCs, cancer stem cells, CMV, *Cytomegalovirus*, EMT, epithelial-mesenchymal transition, GFP, green fluorescent protein, HA, hemagglutinin tag, HCC, hepatocellular carcinoma, HUVECs, human umbilical vein endothelial cells, IRES, internal ribosome entry site, LECT2, leukocyte cell-derived chemotaxin 2, LiAc, lithium acetate, MOI, multiplicity of infection, PD, progressive disease, PI, propidium iodide, qRT-PCR, real-time quantitative polymerase chain reaction, RECIST, response evaluation criteria in solid tumors, rLECT2, recombinant LECT2, SD rat, Sprague Dawley rat, NaAc, sodium acetate, TCGA, the cancer genome atlas, TUNEL, terminal deoxynucleotidyl transferase dUTP nick end labeling, US, ultrasound

## Abstract

Leukocyte cell-derived chemotaxin 2 (LECT2) acts as a tumor suppressor in hepatocellular carcinoma (HCC). However, the antineoplastic mechanism of LECT2, especially its influence on hepatic cancer stem cells (CSCs), remains largely unknown. In The Cancer Genome Atlas cohort, *LECT2* mRNA expression was shown to be associated with stage, grade, recurrence, and overall survival in human HCC patients, and *LECT2* expression was downregulated in hepatoma tissues compared with the adjacent nontumoral liver. Here, we show by immunofluorescence and immunoblot analyses that LECT2 was expressed at lower levels in tumors and in poorly differentiated HCC cell lines. Using functional assays, we also found LECT2 was capable of suppressing oncogenic behaviors such as cell proliferation, anchorage-independent growth, migration, invasiveness, and epithelial-mesenchymal transition in hepatoma cells. Moreover, we show exogenous LECT2 treatment inhibited CSC functions such as tumor sphere formation and drug efflux. Simultaneously, hepatic CSC marker expression was also downregulated, including expression of CD133 and CD44. This was supported by infection with adenovirus encoding LECT2 (Ad-LECT2) in HCC cells. Furthermore, in animal experiments, Ad-LECT2 gene therapy showed potent efficacy in treating HCC. We demonstrate LECT2 overexpression significantly promoted cell apoptosis and reduced neovascularization/CSC expansion in rat hepatoma tissues. Mechanistically, we showed using immunoblot and immunofluorescence analyses that LECT2 inhibited β-catenin signaling *via* the suppression of the hepatocyte growth factor/c-MET axis to diminish CSC properties in HCC cells. In summary, we reveal novel functions of LECT2 in the suppression of hepatic CSCs, suggesting a potential alternative strategy for HCC therapy.

Hepatocellular carcinoma (HCC) is the primary malignancy of liver and constitutes 80 to 90% of all primary liver cancer cases (1). Current HCC treatments include surgical resection, chemotherapy, radiotherapy, immunotherapy, and liver transplantation (2). However, the overall prognosis for advanced HCC remains poor. Sorafenib is a tyrosine kinase inhibitor (TKI) and the first-line drug for unresectable HCC with marginal benefits by extending the median survival time for 3 to 5 months (3). Moreover, sorafenib is frequently associated with adverse side effects, and drug resistance often develops (4). Regorafenib (a TKI) and nivolumab (a PD-1 antibody) are drugs approved for second-line treatment of patients with HCC after sorafenib failure (5, 6). Survival outcomes in patients treated with regorafenib and nivolumab after sorafenib failure is still poor and do not differ significantly (7). Thus, the development of novel therapeutic strategy for HCC treatment is a critical issue.

Leukocyte cell-derived chemotaxin 2 (LECT2) was first identified as a 16-kDa secreted protein from cultured medium of phytohemagglutinin-activated SKW-3 leukemia cells and exhibit chemotaxis for human neutrophils (8). LECT2 is a hepatokine produced by liver, and it plays an important role in the cross-talk of liver and distal organs (9). In addition, the changes in hepatic LECT2 expression are related to acute hepatitis (10), hepatic amyloidosis (11), nonalcoholic fatty liver disease (12), nonalcoholic steatohepatitis (13), liver fibrosis (14), and HCC (15). In liver cancer, constitutively active β-catenin drives LECT2 upregulation in tumor tissues and HCC cells, and LECT2 is a direct target gene of β-catenin in liver (16). Interestingly, LECT2 seems to be a tumor suppressor in liver cancer, and it has been reported that LECT2 inhibits HCC progression mediated the blockade of c-MET signaling (17), tumoral angiogenesis (18), oncogenic behaviors of cancer cells (15) and high-grade inflammation in HCC (19). Although many studies illustrated that LECT2 as a potential tumor suppressor in liver cancer, the detailed antitumor mechanism of LECT2 on HCC, especially hepatic cancer stem cells (CSCs) has remained largely unclear.

CSCs are the most malignant and rare subpopulation of cancer cells involved in tumor initiation, recurrence, metastasis, drug resistance, and neovascularization (20, 21). Epithelial-mesenchymal transition (EMT) is involved in the genesis of CSCs (22). Besides, there are several unique stemness markers such as CD133, CD44, ALDH, EpCAM, and ABCG2, which have been identified and enriched in hepatic CSCs (23). CSCs is considered as one of the promising therapeutic targets for cancer control. In the current study, the biological function of LECT2 in HCC, especially hepatic CSCs, was investigated, and the therapeutic efficacy of intratumoral LECT2 gene delivery by adenovirus (Ad) was evaluated in the immune-competent orthotopic hepatoma model. We demonstrated that LECT2 can inhibit hepatic CSCs expansion by targeting hepatocyte growth factor (HGF)–induced c-MET/GSK3β/β-catenin axis, and this finding provides a novel strategy for HCC management in the future.

## Results

### LECT2 downregulation was correlated with advanced stages and poor prognosis outcome in HCC

**Figure 1:**
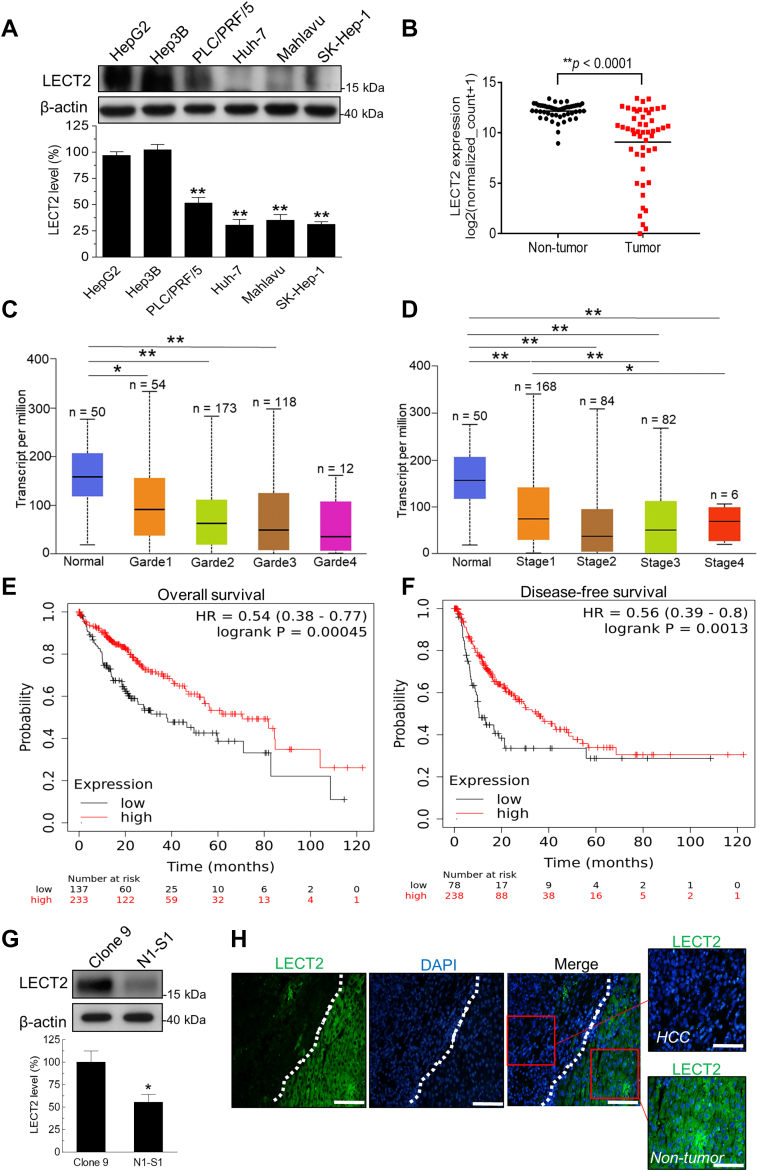
**LECT2 downregulation was correlated with advanced stages and poor prognosis in HCC patients.***A*, immunoblot analysis for LECT2 in HepG2, Hep3B, PLC/PRF/5, Huh-7, Mahlavu, and SK-Hep-1 liver cancer cells. *B*, TCGA analysis for LECT2 mRNA in the paired tumor and nontumor regions of HCC patients (n = 50). *C*, TCGA analysis for LECT2 mRNA expression in human HCC with different grades (grade1 = well differentiated; grade2 = moderately differentiated; grade3 = poorly differentiated; grade4 = undifferentiated). *D*, TCGA analysis for LECT2 mRNA level in human liver cancer with different stages. *E*, Kaplan–Meier analysis for the overall survival in HCC patients with high or low LECT2 expression using TCGA database. *F*, Kaplan-Meier analysis for the disease-free survival in HCC patients with high or low LECT2 level using TCGA database. *G*, immunoblot analysis for LECT2 in N1-S1 rat HCC cells and Clone 9 rat hepatocytes. *H*, immunofluorescent analysis for LECT2 in the tumor and nontumor regions of rat Novikoff hepatoma. Scale bars indicate 200 μm (main panels) and 100 μm (zoom panels). (∗*p* < 0.05, ∗∗*p* < 0.01). TCGA, The Cancer Genome Atlas; HCC, hepatocellular carcinoma; LECT2, leukocyte cell-derived chemotaxin 2.

By using an array of human hepatoma cell lines with different differentiation statuses (24, 25), immunoblot analysis revealed that the LECT2 expression was reduced in the poorly differentiated hepatoma cells than in the well-differentiated ones (Fig. 1*A*). Browsing of The Cancer Genome Atlas (TCGA) data also supported that the LECT2 mRNA level was significantly decreased in HCC tissues compared with nontumor parts (*p* < 0.0001; Fig. 1*B*). Besides, LECT2 downregulation was observed in HCC with high grades (Fig. 1*C*) and advanced stages (Fig. 1*D*). Above all, HCC patients with lower LECT2 level had significantly shorter overall survival (*p* = 0.00045; Fig. 1*E*) and disease-free survival (*p* = 0.0013; Fig. 1*F*). In rat Novikoff hepatoma model, immunoblot analysis showed LECT2 protein level in rat N1-S1 HCC cells was lower than that in rat Clone 9 hepatocytes (Fig. 1*G*). Moreover, LECT2 expression was prominently lower in orthotopic Novikoff hepatoma compared with adjacent nontumor parts (Fig. 1*H*). Together, these results indicate that LECT2 downregulation takes place in human and rat HCC. Moreover, LECT2 downregulation is correlated with malignant progression and worse survival outcome in HCC patients.

### Exogenous LECT2 supply suppressed the oncogenic behaviors and EMT of hepatoma cells

**Figure 2:**
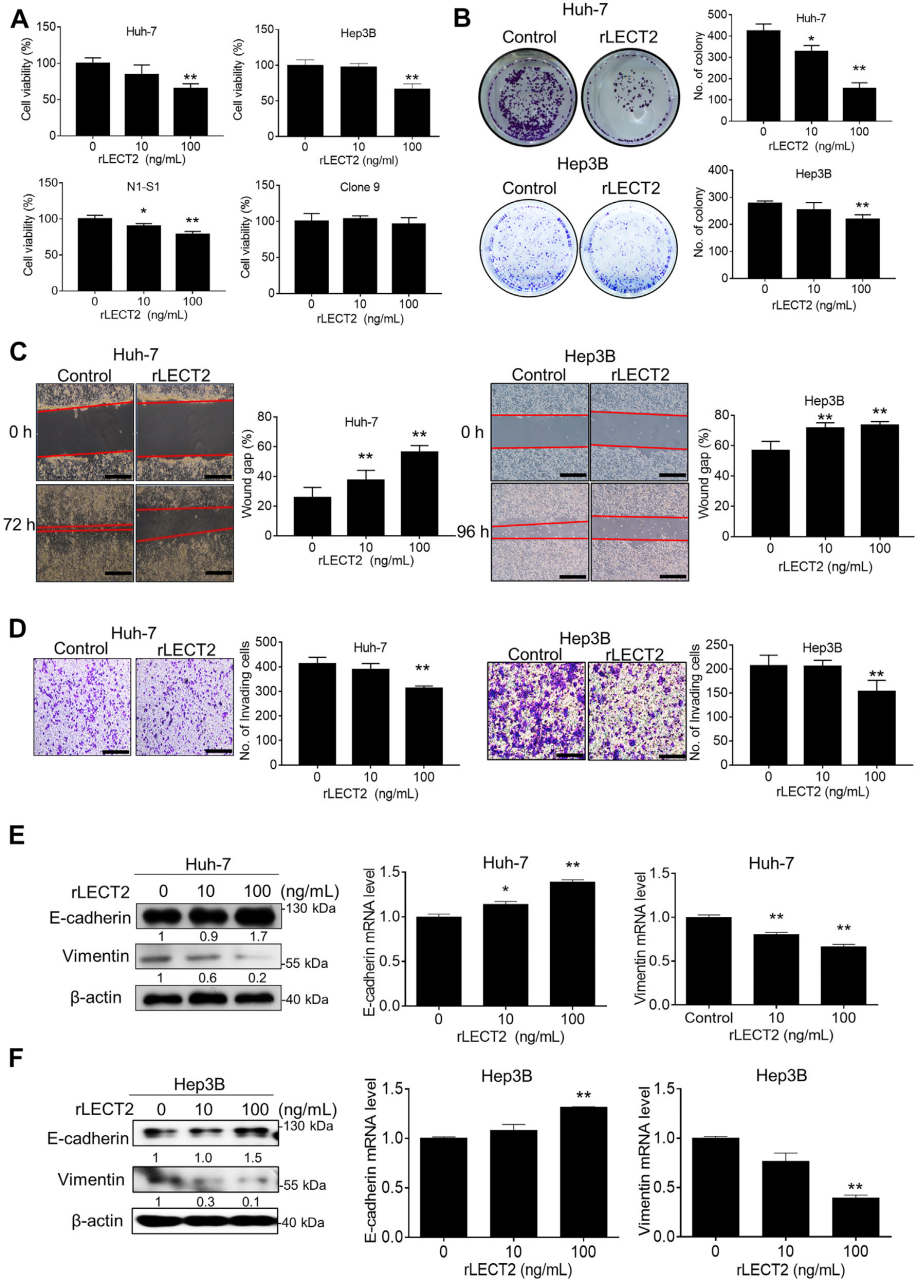
**rLECT2 suppressed oncogenic behaviors and EMT in HCC cells.***A*, Alamar blue assay for cell viability in HCC cell lines (Huh-7, Hep3B and N1-S1) and normal hepatocytes (Clone 9) after rLECT2 treatment (10 and 100 ng/ml) for 48 h. *B*, colony formation assay for anchorage-independent cell growth in Huh-7 and Hep3B cells after rLECT2 treatment (10 and 100 ng/ml) for 10 days. *C*, scratch wound healing assay for cell migration in Huh-7 (from 0 to 72 h) and Hep3B cells (from 0 to 96 h) after rLECT2 treatment (10 and 100 ng/ml). Scale bar = 200 μm. *D*, Boyden chamber assay for cell invasion in Huh-7 and Hep3B cells after rLECT2 treatment (10 and 100 ng/ml) for 24 h. Scale bar = 200 μm. *E*, immunoblot and qRT-PCR analyses for E-cadherin and vimentin in Huh-7 cells after rLECT2 treatment (10 and 100 ng/ml) for 24 h. *F*, immunoblot and qRT-PCR analyses for E-cadherin and vimentin in Hep3B cells after rLECT2 treatment (10 and 100 ng/ml) for 24 h. All data were mean ± SD (∗*p* < 0.05, ∗∗*p* < 0.01). qRT-PCR, real-time quantitative polymerase chain reaction; HCC, hepatocellular carcinoma; rLECT2, recombinant leukocyte cell-derived chemotaxin 2.

Since LECT2 is a secreted factor, we investigated whether exogenous supply of recombinant LECT2 (rLECT2) affected the tumorigenic processess of HCC cells. By using viability assay, it was found that rLECT2 treatment dose-dependently inhibited the proliferation of human hepatoma (Huh-7 and Hep3B) and rat hepatoma (N1-S1) cells (Fig. 2*A*). However, application of rLECT2 did not affect the proliferation of Clone 9 hepatocytes. By using the colony formation assay, exogenous rLECT2 supply significantly suppressed the anchorage-independent growth in Huh-7 and Hep3B cells (Fig. 2*B*). The scratch wound healing assay showed that rLECT2 significantly inhibited the ability of cell migration in Huh-7 and Hep3B cells (Fig. 2*C*). Moreover, the Boyden chamber assay showed that LECT2 significantly suppressed the cell invasiveness in Huh-7 and Hep3B cells (Fig. 2*D*). The effect of LECT2 on EMT of hepatoma cells was further studied in this research. From the immunoblot and qPCR analysis, LECT2 protein treatment significantly upregulated E-cadherin, and downregulated vimentin in Huh-7 and Hep3B cells (Fig. 2, *E* and *F*). Furthermore, rLECT2 not only affected these two EMT markers but also α-SMA and Snail in HCC cells (Fig. S1). These results support the antineoplastic function of LECT2 through suppressing oncogenic behaviors and EMT of HCC cells.

### Intratumor LECT2 gene delivery retarded the progression of established Novikoff hepatoma in rats

**Figure 3:**
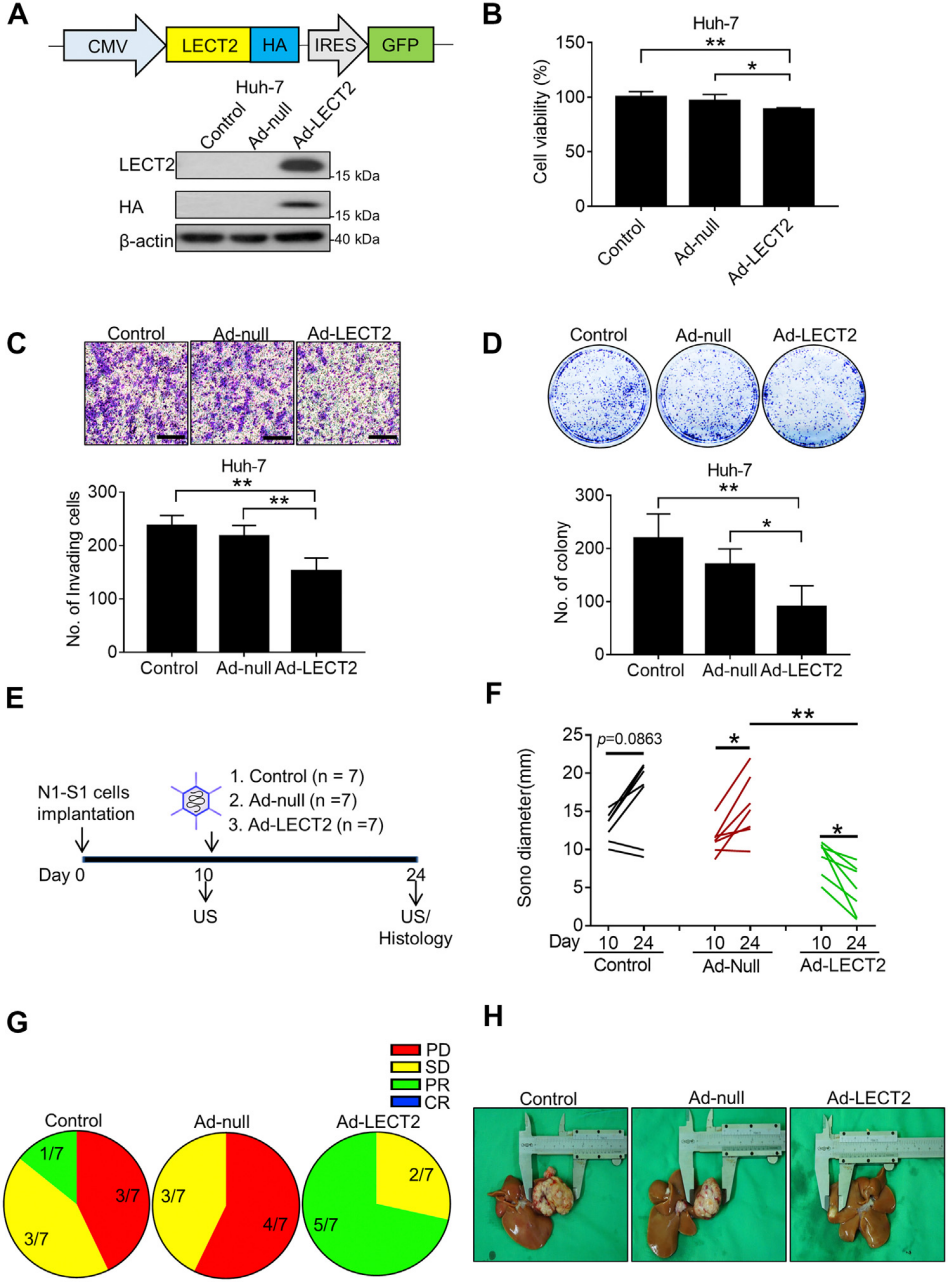
**LECT2 gene delivery by adenovirus inhibited the oncogenic processes of HCC cells and the rat hepatoma progression.***A*, schematic representation of adenovirus vector construct (Ad-LECT2) (upper panel). CMV = *Cytomegalovirus* promoter; HA = hemagglutinin tag; IRES = internal ribosome entry site; GFP = green fluorescent protein. Immunoblot analysis for LECT2 and HA in Huh-7 cells after Ad-LECT2 or Ad-null infection (200 MOI) for 48 h (lower panel). *B*, Alamar blue assay for cell viability in Huh-7 cells after Ad-LECT2 or Ad-null infection (200 MOI) for 48 h. *C*, Boyden chamber assay for cell invasion in Ad-LECT2 or Ad-null-infected (200 MOI for 48 h) Huh-7 cells after seeding for 24 h. Scale bar = 200 μm. *D*, colony formation assay for anchorage-independent cell growth in Ad-LECT2-or Ad-null-infected (200 MOI for 48 h) Huh-7 cells after seeding for 10 days. *E*, experimental scheme of animal study. *F*, US monitoring of rat Novikoff hepatoma before and after therapy. *G*, RECIST analysis for the response of therapy. *H*, photographs of hepatic tumors after animal sacrificing. All data were mean ± SD (∗*p* < 0.05, ∗∗*p* < 0.01). RECIST, Response Evaluation Criteria in Solid Tumors; MOI, multiplicity of infection; HCC, hepatocellular carcinoma; LECT2, leukocyte cell-derived chemotaxin 2; US, ultrasound.

By far, the therapeutic potential of LECT2-based therapy has never been evaluated in animals with orthotopic HCC. For sustained LECT2 production in HCC, we generated a recombinant Ad encoding LECT2 (Ad-LECT2) for gene delivery study in rats bearing established Novikoff hepatoma (Fig. 3*A*). After confirming the transgenes expression in hepatoma cells by immunoblot analysis, the influence of LECT2 gene delivery on the oncogenic behaviors of hepatoma cells was explored. It was found that LECT2 overexpression significantly attenuated the viability (Fig. 3*B*), invasiveness (Fig. 3*C*), anchorage-independent growth (Fig. 3*D*), and cell mobility (Fig. S2) of Huh-7 human HCC cells. Subsequently, we investigated the therapeutic efficacy of LECT2 gene delivery for orthotopic hepatoma in rats (Fig. 3*E*). By implantation of N1-S1 cells at day 0, the established Novikoff hepatoma were detected by ultrasound (US) monitoring at day 10 then administrated with Ad vectors *via* US-guided intratumoral injection. On day 24, the HCC progression in rats receiving various treatment was measured again by US before harvesting hepatoma tissues for histological analysis. Quantification analysis of US-measured hepatoma diameters showed that Ad-LECT2 therapy significantly relieved the tumor burden of HCC (from 9.01 ± 2.25 mm on day 10–4.72 ± 3.15 mm on day 24; n = 7) in rats compared with control (from 12.96 ± 1.94 mm on day 10–16.86 ± 5.17 mm on day 24; n = 7) or Ad-null (from 11.29 ± 1.96 mm on day 10–15.46 ± 4.20 mm on day 24; n = 7) (Fig. 3*F*). Moreover, by the Response Evaluation Criteria in Solid Tumors analysis, none of Ad-LECT2–treated rats had progressive disease (PD), whereas PD was observed in 42.86% (3/7) and 57.14% (4/7) in control and Ad-null group, respectively (Fig. 3*G*). Besides, the dissected HCC tissues was significantly smaller compared with other groups (Fig. 3*H*). These results support the therapeutic potential of LECT2 therapy in halting the progression of existing HCC in immunocompetent rats.

### LECT2 therapy induced cell apoptosis and neovascularization blockade in HCC

**Figure 4:**
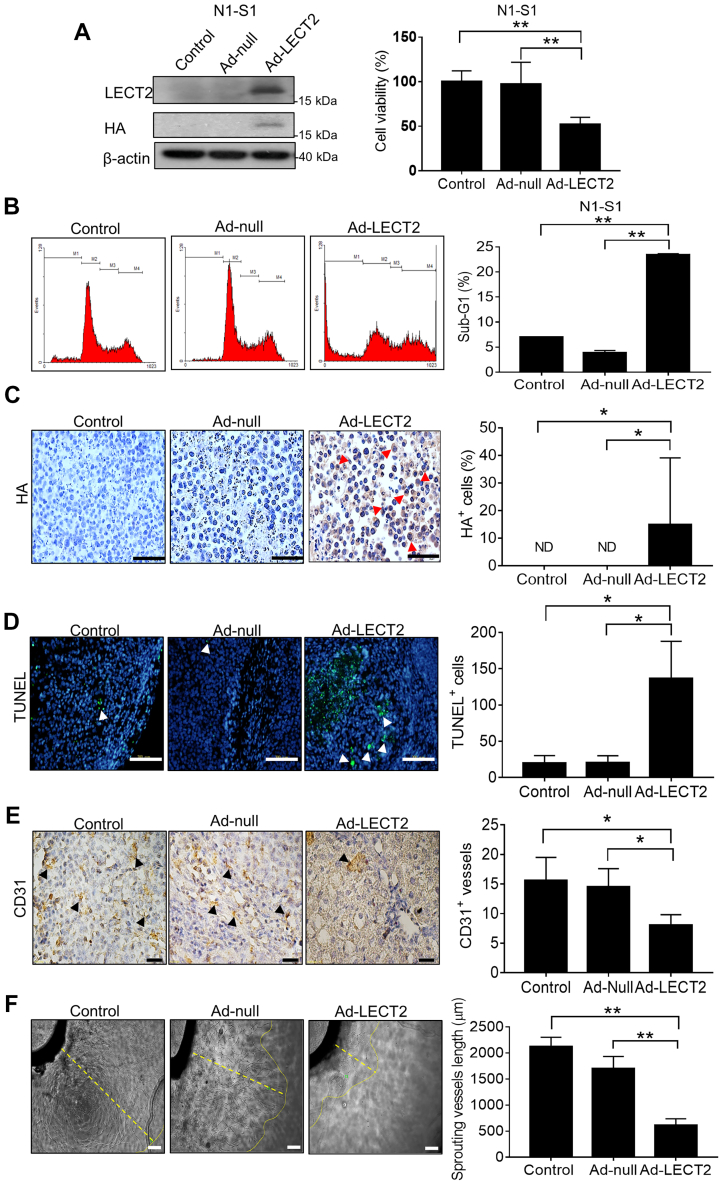
**Ad-LECT2 gene therapy modulated cell apoptosis and neovascularization in HCC.***A*, immunoblot analysis for LECT2 and HA-tag in N1-S1 cells after Ad-LECT2 or Ad-null infection (200 MOI) for 48 h (left panel). Alamar blue assay for cell viability in N1-S1 cells after Ad-LECT2 or Ad-null infection (200 MOI) for 48 h. (right panel). *B*, the sub-G1 fraction of N1-S1 cells after Ad-LECT2 or Ad-null infection (200 MOI) for 48 h was determined by flow cytometry. *C*, immunohistochemistry analysis for HA-tag in rat hepatoma after Ad-null or Ad-LECT2 injection. *Red arrowhead* indicates HA^+^ cells. Scale bar = 200 μm. *D*, TUNEL assay for apoptotic cells in rat hepatic tumor tissues after Ad-null or Ad-LECT2 gene therapy. *White arrowhead* indicates apoptotic cells. Scale bar = 200 μm. *E*, immunohistochemical analysis for CD31^+^ blood vessels in rat HCC tissues after Ad-null or Ad-LECT2 injection. Black arrowhead indicates blood vessels. Scale bar = 100 μm. *F*, rat aortic ring assay for microvessel sprouting after Ad-null or Ad-LECT2 infection (1 x 10^9^ pfu) for 10 days. Scale bar = 200 μm. All data were mean ± SD (∗*p* < 0.05, ∗∗*p* < 0.01). MOI, multiplicity of infection; HCC, hepatocellular carcinoma; LECT2, leukocyte cell-derived chemotaxin 2; HA, hemagglutinin tag; TUNEL, Terminal deoxynucleotidyl transferase dUTP nick end labeling.

Based on the tumor-suppressive function of LECT2 gene delivery by Ad in rat Novikoff HCC model, the effect of Ad-LECT2 infection on cell viability and apoptosis was further investigated in rat N1-S1 cells. By the Alamar blue assay for cell viability, LECT2 gene delivery significantly repressed cell viability (Fig. 4*A*). Moreover, flow cytometry analysis revealed that LECT2 gene delivery significantly increased the ratio of sub-G1 apoptotic cells in cultured N1-S1 hepatoma cells (Fig. 4*B*). For investigating the infection efficiency and specificity of intratumoral Ad-LECT2 injection, the immunohistochemical analysis for hemagglutinin (HA)-tag was performed in this study, and ∼15% HA^+^ cells were shown in the tumor nest of rat HCC (Fig. 4*C*). Besides, the effect of LECT2 gene delivery on apoptosis of HCC was elucidated by terminal deoxynucleotidyl transferase dUTP nick end labeling (TUNEL) staining, and it was shown that Ad-LECT2 administration significantly increased the number of TUNEL^+^ apoptotic cell in rat hepatoma compared with control group (Fig. 4*D*). Since neovascularization blockade frequently leads to apoptosis, the effect of LECT2 gene delivery on angiogenesis of HCC was further studied. By the Panther Pathway and gene enrichment plot analyses using LinkedOmics database, LECT2 expression was negatively correlated with angiogenesis-related genes in human HCC (false discovery rate = 0.14343; ∗∗*p* = 0; normalized enrichment score = −1.5707) (Fig. S3). Based on TCGA analysis, tumor LECT2 expression was significantly lower in HCC patients with vascular invasion (microvascular and macrovascular invasions) than them without vascular invasion (Fig. S4). Thus, the effect of LECT2 gene therapy on tumor vasculature was investigated by histological analysis. It was shown that the CD31^+^ blood vessels were significantly reduced in Ad-LECT2–treated hepatoma compared with control groups (Fig. 4*E*). The antiangiogenic function after LECT2 gene delivery was also evaluated using the aortic ring, Alamar blue, and Boyden chamber assays, which showed that LECT2 overexpression significantly inhibited the microvessel sprouting *ex vivo* (Fig. 4*F*) and the cell functions (cell viability and migration) of human umbilical vein endothelial cells (HUVECs) *in vitro* (Fig. S5*A* and S5*B*). Besides, the TCGA analysis revealed that LECT2 expression was negatively correlated with VEGF (a proangiogenic factor) level in human HCC (∗∗*p* < 0.01) (Fig. S5*C*). From the immunoblot analysis, rLECT2 treatment also inhibited VEGF expression in HCC cells (Fig. S5*D*). Together, these results point out that the inhibition of angiogenesis and the induction of cell apoptosis participate in the anti-HCC function of LECT2.

### Application of rLECT2 inhibited the functions and markers expressions of CSCs in HCC cells

**Figure 5:**
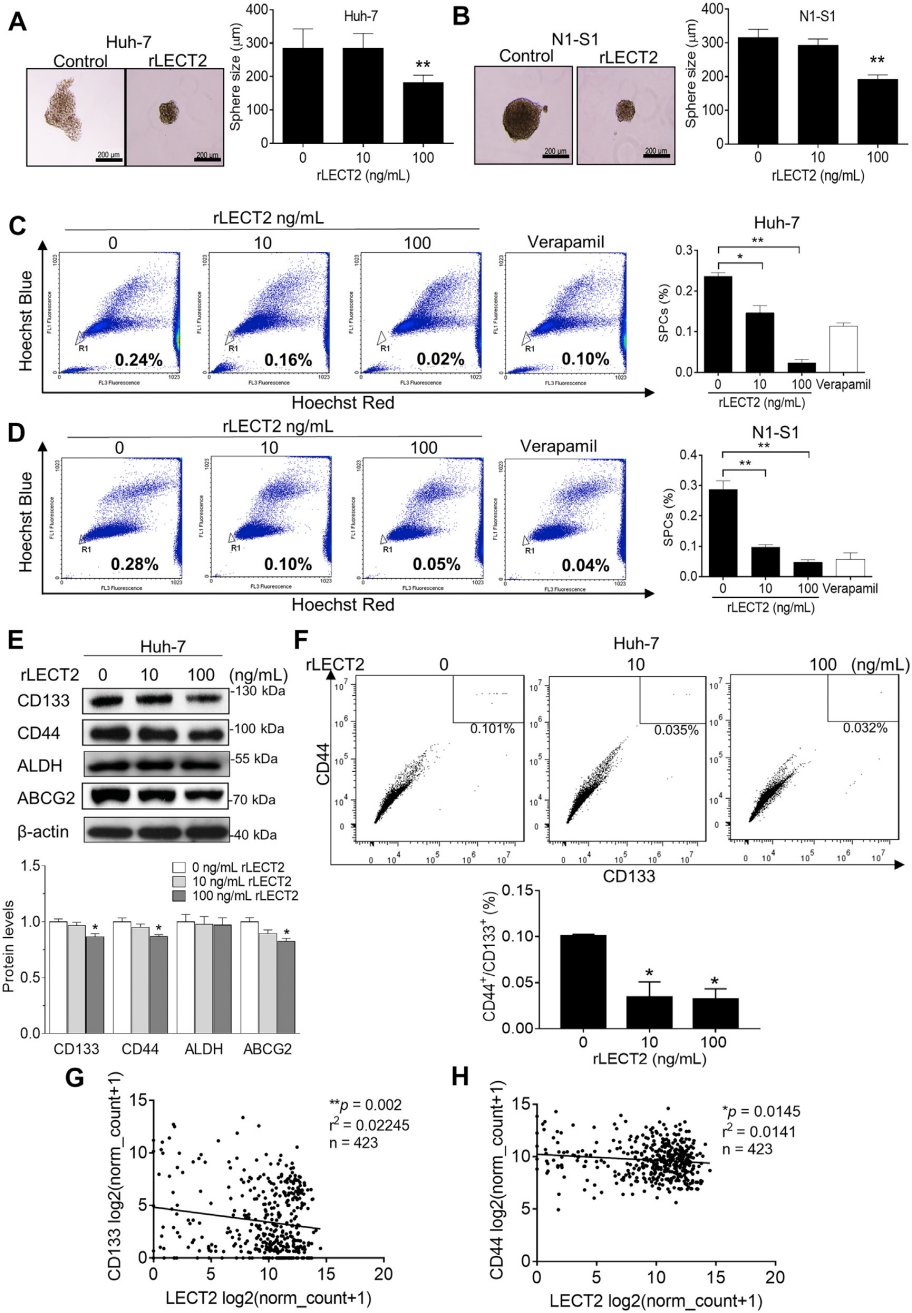
**Exogenous LECT2 inhibited cancer stemness of HCC cells.***A*, tumor sphere assay for cell self-renewal in Huh-7 cells after rLECT2 treatment (10 and 100 ng/ml) for 7 days. Sphere size were analyzed. Scale bar = 200 μm. *B*, tumor sphere assay for cell self-renewal in N1-S1 cells after rLECT2 treatment (10 and 100 ng/ml) for 7 days. Sphere size was analyzed. Scale bar = 200 μm. *C*, side population cells assay for drug efflux in Huh-7 cells after rLECT2 treatment (10 and 100 ng/ml) for 24 h. *D*, side population cells assay for drug efflux in N1-S1 cells after rLECT2 treatment (10 and 100 ng/ml) for 24 h. *E*, immunoblot analysis for CD133, CD44, ALDH, and ABCG2 in Huh-7 cells after rLECT2 treatment (10 and 100 ng/ml) for 24 h. The experiments of Fig. 5*E* and Fig. 7*F* were performed using the same sample and blot, and the identical β-actin bands are shown in these two panels. *F*, flow cytometry analysis for CD44^+^/CD133^+^ CSCs in Huh-7 cells after rLECT2 (10 and 100 ng/ml) treatment for 24 h. *G*, TCGA analysis for the correlation between LECT2 and CD133 expressions in HCC patients. *H*, TCGA analysis for the correlation between LECT2 and CD44 levels in HCC patients. (∗*p* < 0.05, ∗∗*p* < 0.01). TCGA, The Cancer Genome Atlas; HCC, hepatocellular carcinoma; LECT2, leukocyte cell-derived chemotaxin 2; rLECT2, recombinant LECT2.

Because LECT2 elicits the reversal of EMT, which is a prerequisite to cancer stemness (22). Thus, we analyzed the effect of rLECT2 on the functions of CSC, including self-renewal and drug efflux. By sphere formation assay, it was found that exogenous rLECT2 treatment significantly and dose-dependently reduced the sphere size in Huh-7 cells (Fig. 5*A*) and N1-S1 cells (Fig. 5*B*). Similarly, by using side population (SP) analysis, rLECT2 supply significantly reduced the ratio of drug-pumping SP cells (SPCs) in Huh-7 (Fig. 5*C*) and N1-S1 cells (Fig. 5*D*). After validating the CSC-regulating function, we examined whether exogenous LECT2 modulated the expression of hepatic CSCs markers in HCC cells by immunoblot analysis. Application of rLECT2 induced a dose-dependent reduction in CD133, CD44, and ABCG2 protein levels of Huh-7 (Fig. 5*E*) and N1-S1 cells (Fig. S6). Reportedly, hepatic CSCs and HCC cell origin could be precisely defined by co-expression of CD44 and CD133 cell surface markers (26). By flow cytometry analysis for hepatic CD44^+^/CD133^+^ CSCs, rLECT2 treatment dose-dependently and significantly deprived CD44^+^/CD133^+^ subpopulation in hepatoma cells (Fig. 5*F*). Interestingly, the TCGA analysis revealed that LECT2 expression was negatively correlated with CD133 (∗∗*p* < 0.01; Fig. 5*G*) or CD44 (∗*p* < 0.05; Fig. 5*H*) level in human HCC. These findings strongly advocate the anti-CSCs potential of LECT2.

### LECT2 gene therapy mitigated the CD44/CD133 expression and CSCs expansion in HCC *in vitro* and *in vivo*

**Figure 6:**
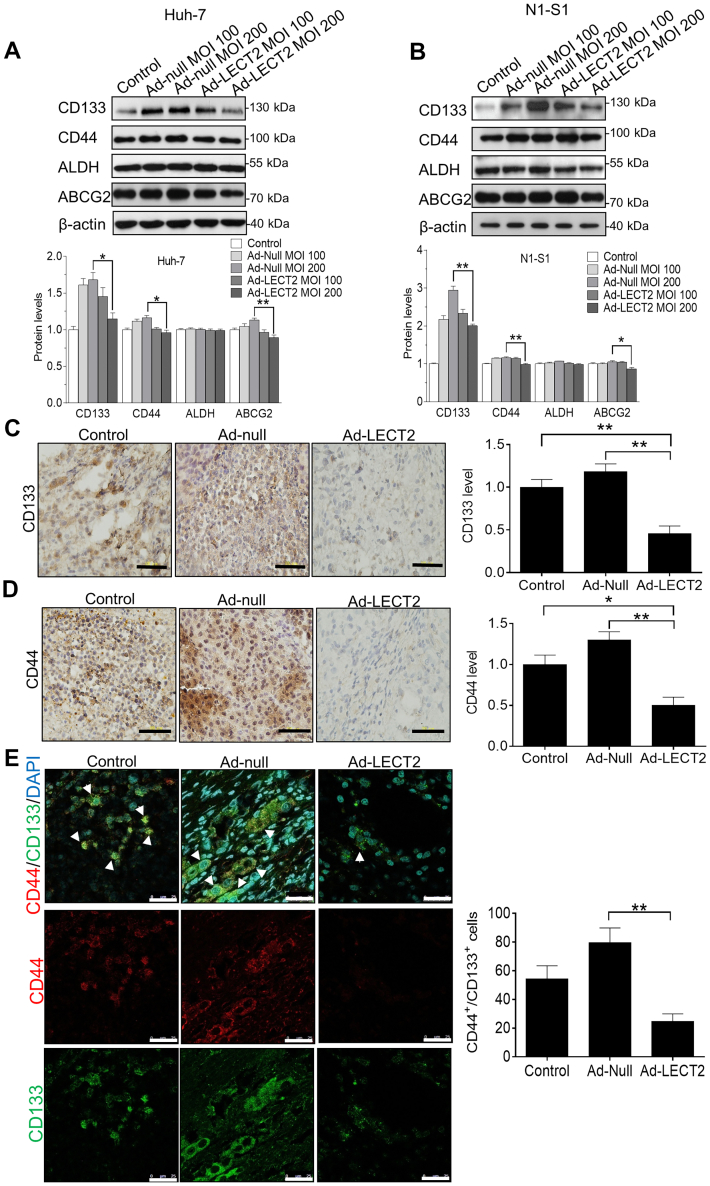
**Gene delivery by adenovirus repressed the expression of CSCs markers in HCC cells and rat liver tumor.***A*, immunoblot analysis for CD133, CD44, ALDH, and ABCG2 in Huh-7 cells after Ad-LECT2 or Ad-null infection (100 and 200 MOI) for 48 h. *B*, immunoblot analysis for CD133, CD44, ALDH, and ABCG2 in N1-S1 cells after Ad-LECT2 or Ad-null infection (100 and 200 MOI) for 48 h. *C*, immunohistochemical analysis for CD133 in rat liver cancer after Ad-null or Ad-LECT2 injection. Scale bar = 200 μm. *D*, immunohistochemical analysis for CD44 in rat hepatoma after Ad-null or Ad-LECT2 injection. Scale bar = 200 μm. *E*, immunofluorescent analysis for CD44^+^/CD133^+^ hepatic CSCs in rat HCC after Ad-null or Ad-LECT2 gene therapy. Scale bar = 200 μm. All data were mean ± SD (∗*p* < 0.05, ∗∗*p* < 0.01). HCC, hepatocellular carcinoma; LECT2, leukocyte cell-derived chemotaxin 2; CSCs, cancer stem cells; MOI, multiplicity of infection.

Subsequently, we elucidated whether LECT2 gene delivery attenuated the expression of CSCs markers in HCC cells and in hepatoma tissues. By immunoblot analysis, it was shown Ad-mediated LECT2 overexpression significantly downregulated the CD133, CD44, and ABCG2 expression in Huh-7 cells (Fig. 6*A*) and N1-S1 cells (Fig. 6*B*), which were consistent with results from rLECT2 treatment. Likewise, immunohistochemical analysis showed that intratumoral LECT2 gene delivery significantly inhibited the expression of CD133 (Fig. 6*C*) and CD44 (Fig. 6*D*) in Novikoff hepatoma tissues. Since CD44^+^/CD133^+^ cells account for an important subpopulation of hepatic CSCs (26), immunofluorescent analysis further confirmed that LECT2 therapy significantly decreased the CD44^+^/CD133^+^ hepatic CSCs in Novikoff hepatoma (∗∗*p* < 0.01; Fig. 6*E*). These results strongly support that intratumoral LECT2 gene therapy reduce the expansion of hepatic CSCs *in vitro* and *in vivo*.

### LECT2 repressed the stemness-modulating β-catenin signaling in liver cancer cells and hepatoma

**Figure 7:**
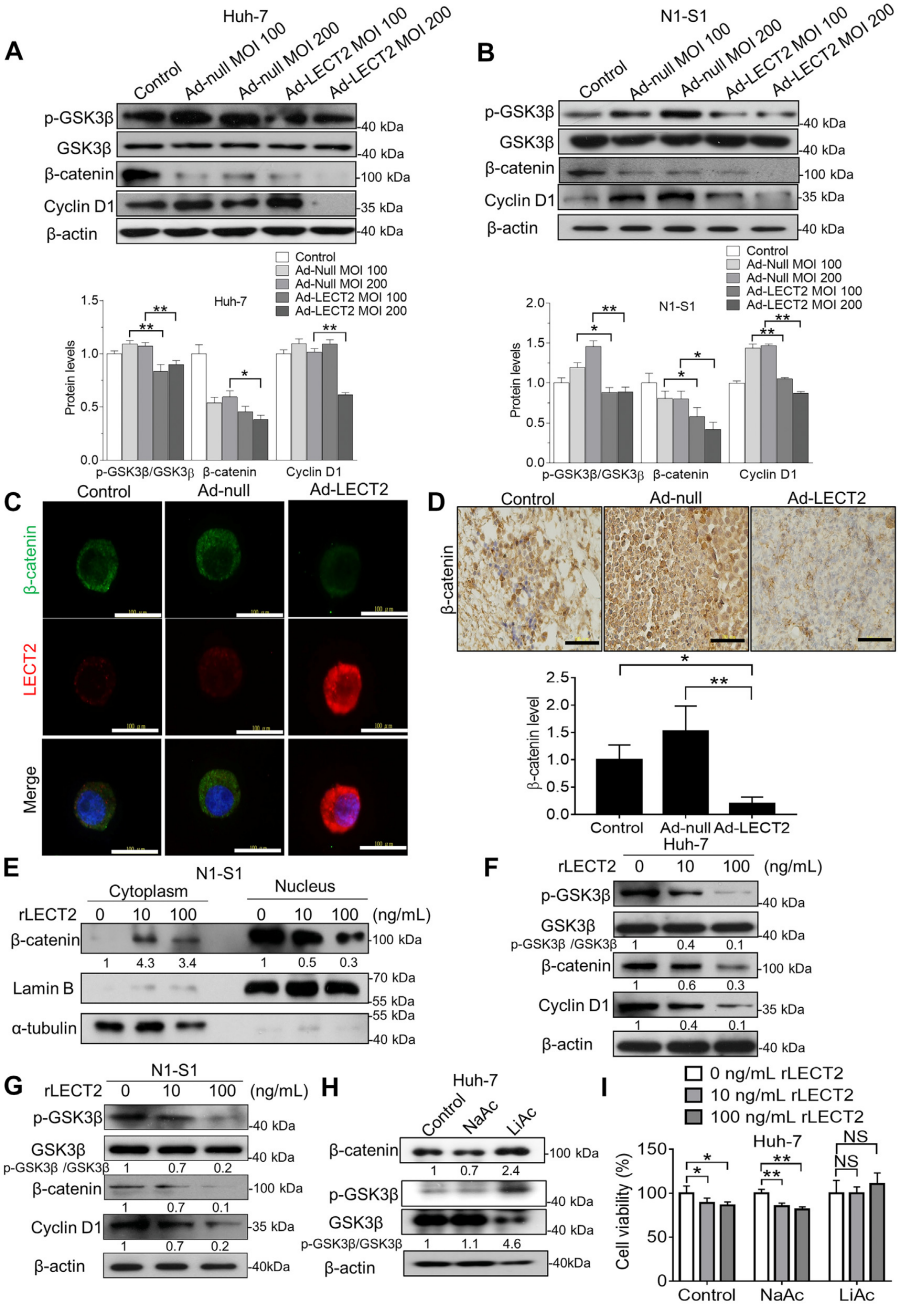
**LECT2 inhibited β-catenin signaling in HCC cells and rat hepatoma.***A*, immunoblot analysis for p-GSK3β, GSK3β, β-catenin, and Cyclin D1 in Huh-7 cells after Ad-LECT2 or Ad-null infection (100 and 200 MOI) for 48 h. *B*, immunoblot analysis for p-GSK3β, GSK3β, β-catenin, and Cyclin D1 in N1-S1 cells after Ad-LECT2 or Ad-null infection (100 and 200 MOI) for 48 h. *C*, immunofluorescent analysis for β-catenin in N1-S1 cells after Ad-LECT2 or Ad-null infection (200 MOI) for 48 h. Scale bar = 100 μm. *D*, immunohistochemical analysis for β-catenin in rat hepatoma after Ad-null or Ad-LECT2 administration. Scale bar = 100 μm. *E*, immunoblot analysis for cytosolic and nuclear β-catenin in N1-S1 cells after rLECT2 treatment (10 and 100 ng/ml) for 24 h. *F*, immunoblot analysis for p-GSK3β, GSK3β, β-catenin, and Cyclin D1 in Huh-7 cells after rLECT2 treatment (10 and 100 ng/ml) for 24 h. The experiments of Fig. 5*E* and Fig. 7*F* were performed using the same sample and blot, and the identical β-actin bands are shown in these two panels. *G*, immunoblot analysis for p-GSK3β, GSK3β, β-catenin, and Cyclin D1 in N1-S1 cells after rLECT2 treatment (10 and 100 ng/ml) for 24 h. The experiments of Figs. 7*G* and S6 were performed using the same sample and blot, and the identical β-actin bands are shown in these two panels. *H*, immunoblot analysis for β-catenin, p-GSK3β, and GSK3β in Huh-7 cells after 20 mM NaAc (a vehicle control) or LiAc (GSK3β inhibitor) for 48 h. *I*, Alamar blue assay for cell viability in NaAc-treated or LiAc-treated Huh-7 cells after rLECT2 (10 and 100 ng/ml) treatment for 48 h. All data were mean ± SD (∗*p* < 0.05, ∗∗*p* < 0.01, NS = not significant). NaAc, sodium acetate; LiAc, lithium acetate; HCC, hepatocellular carcinoma; LECT2, leukocyte cell-derived chemotaxin 2; MOI, multiplicity of infection.

By the Panther Pathway (Fig. S3*A*) and gene enrichment plot (Fig. S7) analyses using LinkedOmics database, LECT2 expression was negatively correlated with Wnt/β-catenin signaling pathway in human HCC (false discovery rate = 0.17576; ∗∗*p* = 0; normalized enrichment score = −1.5048). GSK3β/β-catenin signaling plays an important role in maintenance of CD44^+^ and CD133^+^ hepatic CSCs (27, 28), and CD44/p-GSK3β/β-catenin were upregulated in CD133^+^ N1-S1 cells compared with CD133^−^ N1-S1 cells (Fig. S8*A*). Besides, β-catenin overexpression by lithium (a GSK3β inhibitor) upregulated CD133 and CD44 (Fig. S8*B*), and quercetin (a β-catenin inhibitor) caused CD44/CD133 downregulation in N1-S1 cells (Fig. S8*C*). Reportedly, β-catenin signaling also participates in EMT and VEGF production of cancer cells (29, 30). By the immunoblot analysis, lithium-induced β-catenin overexpression also promoted E-cadherin loss, vimentin upregulation, and VEGF production in Huh-7 cells (Fig. S8*D*). Thus, we employed the immunoblot analysis to delineate whether LECT2-modulated cancer stemness/EMT/VEGF pathways is mediated GSK3β/β-catenin signaling. In Huh-7 and N1-S1 cells, LECT2 gene delivery significantly attenuated the phosphorylation at Ser9 of GSK3β, the expression of β-catenin and cyclin D1, one of β-catenin target genes (Fig. 7, *A* and *B*). Consistently, immunofluorescent analysis showed that LECT2 gene delivery significantly mitigated the β-catenin expression in N1-S1 cells (Fig. 7*C*). Above all, immunohistochemical analysis confirmed that Ad-LECT2 therapy significantly diminished the β-catenin level in rat hepatoma tissues (∗∗*p* < 0.01; Fig. 7*D*). Because nuclear entry is essential to the transcriptionally regulatory function of β-catenin, we studied the effect of exogenous rLECT2 supply on nuclear translocation of β-catenin in HCC cells by subcellular fractionations and immunoblot analysis. It was observed that rLECT2 treatment decreased the β-catenin level in the nucleus of N1-S1 cells (Fig. 7*E*). Moreover, application of rLECT2 perturbed the GSK3β phosphorylation and β-catenin/cyclin D1 expression in Huh-7 cells (Fig. 7*F*) and N1-S1 cells (Fig. 7*G*). Finally, the Alamar blue analysis revealed that β-catenin restoration by lithium treatment blocked the antiproliferative effect of LECT2 in Huh-7 cells (Fig. 7, H and I). Together, LECT2 may antagonize β-catenin signaling, thereby suppressing cancer stemness/EMT/angiogenesis in HCC. Besides, this finding is consistent with a previous report that exogenous LECT2 could suppressed Wnt/β-catenin signaling in colon (31).

### LECT2 antagonized the basal and HGF-stimulated c-MET/GSK3β/β-catenin axis in liver cancer cells

**Figure 8:**
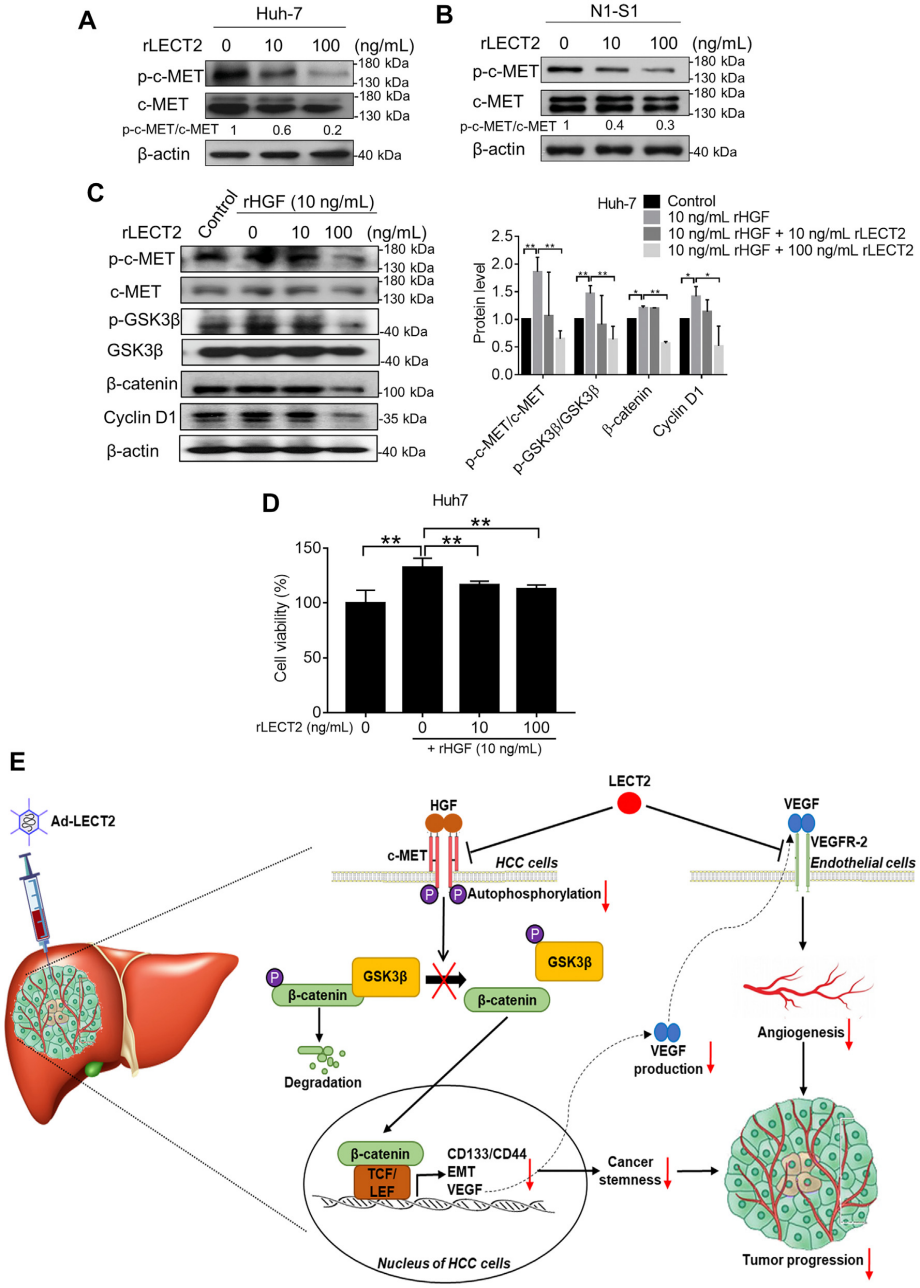
**Exogenous LECT2 antagonized HGF-promoted c-MET/GSK3β/β-catenin axis in HCC cells.***A*, immunoblot analysis for p-c-MET and c-MET in Huh-7 cells after rLECT2 treatment (10 and 100 ng/ml) for 24 h. *B*, immunoblot analysis for p-c-MET and c-MET in N1-S1 cells after rLECT2 treatment (10 and 100 ng/ml) for 24 h. *C*, immunoblot analysis for p-c-MET, c-MET, p-GSK3β, GSK3β, β-catenin, and Cyclin D1 in Huh-7 cells after rHGF (10 ng/ml) ± rLECT2 (10 or 100 ng/ml) treatment for 24h. *D*, Alamar blue assay for cell viability in Huh7 cells after rHGF (10 ng/ml) ± rLECT2 (10 or 100 ng/ml) treatment for 24h. *E*, hypothetical model for anti-HCC mechanism of LECT2. Data were mean ± SD (∗*p* < 0.05, ∗∗*p* < 0.01). LECT2, leukocyte cell-derived chemotaxin 2; HGF, hepatocyte growth factor; HCC, hepatocellular carcinoma.

c-MET activation is known to influence the Wnt/β-catenin signaling and promote the subsequent phosphorylation at Ser9 and inactivation of GSK3β (32). It has been reported that LECT2 acts as a potent antagonist of c-MET signaling in many cancer types (17, 33, 34) Thus, we investigated whether exogenous LECT2 affected the c-MET signaling in HCC cells. Immunoblot analysis showed that rLECT2 treatment dose-dependently inhibited the c-MET autophosphorylation at Tyr1234/1235 in Huh-7 cells (Fig. 8*A*) and N1-S1 cells (Fig. 8*B*). In the presence of c-Met ligand HGF, rLECT2 treatment remained capable of inhibiting the HGF-induced c-MET/GSK3β/β-catenin/cyclin D1 axis in Huh-7 cells (Fig. 8*C*). Moreover, the Alamar blue assay revealed that rLECT2 treatment partially inhibited HGF-stimulated cell proliferation in Huh-7 cells (Fig. 8*D*), and this indicates that blocking of HGF/c-MET signaling partially participates in the antiproliferative effect of LECT2 in HCC cells. Besides, the activities of c-MET and β-catenin signaling were not significantly and negatively correlated with the differentiation status and LECT2 expression in liver cancer cells (Fig. S9) (Fig. 1*A*). From the immunoblot analysis, c-MET inhibitor (XL184) treatment repressed β-catenin signaling, EMT, and VEGF expression in Huh-7 cells (Fig. S10*A*), and c-MET inhibition also caused CD44/CD133 downregulation and the suppression of cell self-renewal in Huh-7 cells (Fig. S10*B* and S10*C*). Interestingly, LECT2 inhibited c-MET/β-catenin axis in both CD133^−^ and CD133^+^ fractions of Huh-7 cells (Fig. S11), and this indicated that anticancer effect of LECT2 is not specifically acted on CSCs. However, the blocking of HGF/c-MET/β-catenin pathway was involved in the LECT2-induced anti-HCC function.

## Discussion

HCC is a malignant neoplasm with a high morbidity and mortality rate. One of the influencing factors for the failure of HCC therapy is the emergence of CSCs, which conferred resistance to current treatment of doxorubicin and sorafenib (35, 36). Although novel therapies such as the second generation TKIs and immunotherapies have offered additional therapeutic landscape (37), it is pivotal to identify and develop the CSCs-targeting modalities for HCC control. The earlier studies by different groups have collectively pointed out that LECT2 could be a promising molecular target for HCC with antiangiogenic, antitumorigenic, and immunemodulating potency (15, 17, 18, 19, 38). However, the diagnostic and therapeutic potentials of LECT2 have not been systematically characterized. In the present study, we have employed various approaches, including the TCGA bioinformatics analysis, the array of human HCC cell lines, and orthotopic HCC model in animals, to unambiguously demonstrate that LECT2 loss is involved in liver carcinogenesis. Importantly, LECT2 deficiency is associated with advanced grades/stages and correlated with worse survival outcome in HCC patients, indicating that LECT2 may constitute a promising prognostic factor. Furthermore, the present study has also demonstrated that LECT2 restoration either by recombinant protein supply or gene delivery is effective in perturbing the HCC progression *in vitro* and *in vivo*, advocating LECT2-based therapy may facilitate a novel CSCs-targeting therapy or serve as an adjuvant therapy in conjunction with current HCC therapies for HCC control.

A previous study mentioned that LECT2 inhibited not only the cell migration and invasion in human HCC cell lines but also the tumor growth in the xenograft animal model (15). In this study, rLECT2 treatment or Ad-LECT2 infection reduced cell viability in rat and human HCC cells but not affected the cell growth in normal hepatocytes. This result represents that LECT2-based HCC therapy shows high specificity toward hepatoma cells. Furthermore, exogenous LECT2 or Ad-LECT2 administration effectively suppressed anchorage-independent growth, cell migration, and cell invasiveness companied with EMT inhibition in both rat and human hepatoma cells. Together, LECT2 functions a potent tumor suppressor in HCC. Importantly, LECT2 is identified as an endogenous inhibitor of CSCs. Exogenous LECT2 potentially repressed CSCs properties such as cell self-renewal and drug efflux in hepatoma cells accompanied with the downregulation of CSCs markers. This finding is similar to previous study indicated that poorly differentiated tumor nodules inducing in LECT2-deficient HCC have a stemness/EMT/metastasis signature (38). CSCs play an important role in chemoresistance and resistance to TKIs (such as sorafenib) in HCC (23). At present, there is no drug targeting at CSCs available for advanced HCC. Therefore, anti-CSCs ability of LECT2 implicates that LECT2-based therapy may constitute an adjuvant therapy in conjunction with the current first-line HCC drugs such as doxorubicin and sorafenib. In the animal experiment, we demonstrated that Ad-LECT2 gene therapy showed excellent therapeutic efficacy in immune-competent orthotopic hepatoma. By the histological analysis, hepatic CSCs markers CD133 and CD44 were downregulated accompanied with the induction of cell apoptosis and the blockade of neovascularization in HCC tissues after Ad-LECT2 gene therapy, and it has been reported that LECT2 can antagonize VEGFR2 receptor activation to inhibit angiogenesis in HCC (18). In our previous study, this immune-competent orthotopic HCC model can reflect the truest experimental results and mimic the clinical condition (39), and this HCC model is a powerful tool for the study of tumor microenvironment and cancer immunology in liver cancer. It has been reported that LECT2 is an important interconnected modulator of liver β-catenin-induced inflammation (19), and LECT2 ablation worsened the formation of highly malignant HCC with lung metastasis under β-catenin–induced liver inflammation. Moreover, LECT2 deletion promotes the infiltration of inflammatory and immature monocytes with immunosuppressive capacities and tumor-promoting potential (38). In our further research, the effect of LECT2 on cancer immune should be investigated in this immune-competent orthotopic HCC model.

In the mechanistic study, we herewith proposed a mechanistic model for LECT2-mediated HCC suppression (Fig. 8*E*). HGF/c-MET interaction can promote the phosphorylation and inactivation of GSK3β, and then, the loss function of GSK3β causes the stabilization and nuclear localization of β-catenin. Subsequently, β-catenin forms complex with TCF or LEF to modulate the gene transcription of CSCs/EMT markers and VEGF (29, 30, 40). Reportedly, LECT2 binds to the α-chain of c-MET and inhibits HGF-induced c-MET autophosphorylation at Tyr1234/1235 by recruiting protein tyrosine phosphatase 1B (PTP-1B) (17), and we demonstrated that LECT2 restoration antagonizes the HGF/c-MET signaling as well as its downstream GSK3β/β-catenin axis, thereby eliciting downregulation of CSCs-related genes, EMT reversal, and reduction of VEGF expression. In addition to antineoplastic function, LECT2 repressed the production of proangiogenic factor VEGF by blocking HGF-induced c-MET/GSK3β/β-catenin axis, and it has been reported that LECT2 also inhibits VEGFR2 phosphorylation by directly binding to the extracellular domain (1–746 amino acids) of VEGFR2 (18).

In summary, this preclinical study reveals that intratumoral LECT2 therapy seems well tolerated and effectively abrogates the progression of established HCC in immunocompetent animals through inhibition of angiogenesis and cancer stemness. These promising data support the antineoplastic potential of LECT2 therapy for control of malignant HCC. However, there are still some limitations and concerns for the translational application of LECT2 therapy. For example, this study did not explore whether LECT2 therapy extended the life span of rats bearing advanced HCC. Besides, excessive LECT2 production in the liver might result in undesired metabolic consequence. It has been reported high fatty diet induces inhibition of AMPK pathway and hepatic LECT2 overproduction, which increases the circulating LECT2 to promote insulin resistance in muscle (41). Moreover, abnormal LECT2 deposition in the liver or kidney is associated with the hepatic or renal amyloidosis, respectively (11, 42). In the present study, hepatic amyloidosis was not found in the nontumor section of hepatoma after Ad-LECT2 therapy. Besides, any abnormalities including weight loss, accidental mortality, and behavior changes were not observed. These findings implicate that intratumor LECT2 gene therapy seemed well tolerated and safe in HCC-bearing rats. Nevertheless, future studies are warranted to extensively evaluate the potential of LECT2-based therapy for HCC management and its probable long-term adverse effect. In this study, it seems to Ad-null infection slightly caused the upregulation of β-catenin, CD133, and CD44 in rat HCC. Reportedly, adenoviral vector can induce Akt activation in host cells (43), and Akt phosphorylation can increase the stability and nuclear localization of β-catenin by inactivating GSK3β (44). Subsequently, the activation of β-catenin signaling may promote CD44/CD133 upregulation (27, 28). Nevertheless, adenoviral vector has been applied in the clinical use such as Covid-19 vaccine (45).

## Experimental procedures

### Cell culture and drugs

Clone 9 rat hepatocytes, N1-S1 rat HCC cells, and HUVECs were purchased from the Food Industry Research and Development Institute. HepG2, Hep3B, PLC, Huh7, Mahlavu, and SK-Hep-1 Human HCC cells were purchased from the American Type Culture Collection with validated STR-PCR profile. Clone 9 cells cultured in F12-K medium (Gibco) containing 10% fetal calf serum (HyClone). N1S1 cells were maintained in RPMI-1640 medium (Gibco) containing 10% calf serum (HyClone). HepG2, Hep3B, PLC, Huh7, Mahlavu, and SK-Hep-1 human hepatoma cells were cultured in Dulbecco's modified Eagle's medium (Gibco) containing 10% calf serum (HyClone). HUVECs (passage: 3–6) were cultured in M199 medium (Life Technologies) containing 15% fetal calf serum, 20 U/ml porcine heparin (Sigma), and 100 g/ml endothelial cell growth supplement (Calbiochem). All the media for cell culture were supplemented with 2 mM L-glutamine (HyClone), 100 mg/ml streptomycin (HyClone), and 100 U/ml penicillin (HyClone). All cells were maintained under humidified conditions in 95% air and 5% CO_2_ at 37 °C. Recombinant HGF (H5791), sodium acetate, lithium acetate (a GSK3β inhibitor) (46) and quercetin (β-catenin inhibitor) (47) were purchased from Sigma-Aldrich. XL184 (c-MET inhibitor) (48) was purchased from TargetMol.

### Production of recombinant LECT2

The human LECT2 complementary DNA (cDNA) was constructed into pET15b vector (Novagen) and transformed into BL-21 cells (DE3, pLysS; Novagen). After induction, the 6x-histidine-tagged HDGF protein was purified on an NTA-agarose affinity column (Qiagen) and desalted on a G25 Sephadex column (Amersham Pharmacia). The recombinant protein was passed through Detoxi-Gel (Pierce Biotechnology) to minimize contamination by endotoxin.

### Generation of adenoviral vectors carrying LECT2 (Ad-LECT2)

The full length human LECT2 cDNA were subcloned into Ad transfer vectors, pShuttle-IRES-hfGFP with HA tag (Stratagene). The vectors carrying full length LECT2 were cotransfected into 293 cells, a plasmid containing the entire type5 Ad genome with E1-insertion and E3 deletion by calcium phosphate protocol to generate recombinant virus through homologous recombination. The virus plaques are picked and verified by checking cytopathic effect, PCR, and Western blot prior to amplification. The virus is amplified in 293 cells, purified by two rounds of cesium chloride gradient ultracentrifugation, and dialyzed against buffer containing 10 mM Tris, pH 7.5, 1 mM MgCl_2_, and 10% glycerol at 4 °C. The titer of virus solution was determined by measuring absorbance at wavelength of 260 nm and plaque forming assay in 293 cells before storage at −80 °C.

### Animal experiments

All experimental procedures were reviewed and approved by the Institutional Animal Care and Use Committee at National Sun Yat-sen University (IACUC Approval No. 10716). The US-guided induction of Novikoff hepatoma in Sprague Dawley (SD) rats was performed as previously described (49). Briefly, SD rats (male, 6 weeks old) were implanted with N1-S1 cells by US-guided injection on day 0. After confirming the HCC formation (with diameter larger than 5 mm) by US monitoring on day 10, rats (n = 21) were randomly and double-blindly divided into three groups receiving the following treatment *via* US-guided intratumoral injection: [1] saline (100 μl; n = 7), [2] Ad-null (5 × 10^9^ plaque-forming unit (pfu) in 100 μl saline) by intratumoral injection; n = 7), and [3] Ad-LECT2 (5 × 10^9^ pfu in 100 μl saline). After gene therapy for 14 days, the tumor size was followed by US. Based on the tumor diameters measured by US before and after various therapies, the disease status in animals was evaluated according to Response Evaluation Criteria in Solid Tumors, ver.1.1 (50). In brief, rats with an increase of 20% or more in tumor size or those with new tumors were regarded as having PD. Rats with a change in tumor size ranging from an increase of < 20% to a decrease of <30% and with no new tumor were stratified as having stable disease. Rats with a 30% or greater decrease in the target tumor were regarded as achieving partial response. Rats with disappearance of the tumor were stratified as achieving complete response.

### Immunoblot analysis

The preparation of cell lysates and cell fractions was performed as previously described (51). The protein concentration was determined by the BCA protein assay kit (Pierce Biotechnology). SDS-PAGE and immunoblot were performed as described previously (52). LECT2 (sc-47101, 0.4 μg/ml), CD133 (sc-30219, 0.4 μg/ml), CD44 (sc-9960, 0.4 μg/ml), ABCG2 (sc-130933, 0.4 μg/ml), ALDH (sc-166362, 0.2 μg/ml), E-cadherin (sc-7870, 0.4 μg/ml), vimentin (sc-32322, 0.4 μg/ml), β-catenin (sc-7199, 0.2 μg/ml), p-c-MET (Try1234/1235) (sc-101736, 0.4 μg/ml), c-MET (sc-161, 0.4 μg/ml), p-GSK3β (Ser9) (sc-11757, 0.4 μg/ml), GSK3β (sc-9166, 0.4 μg/ml), cyclin D1 (sc-20044, 0.4 μg/ml), lamin B (sc-6216, 0.4 μg/ml), α-tubulin (sc-8035, 0.2 μg/ml), VEGF (sc-152, 0.4 μg/ml), and HA-tag (sc-7392, 0.4 μg/ml) (Santa Cruz Biotechnology) and β-actin (A5441, 0.2 μg/ml) (Sigma-Aldrich) primary antibodies were used in this study.

### Real-time quantitative RT-PCR

RNA extraction and cDNA synthesis were performed as previously described (53), and cDNA was used as template for real-time quantitative RT-PCR analysis. Amplification and detection were performed by SYBRR Green master mixes (Thermo Fisher Scientific) in Applied Biosystems 7500 Fast Real-Time PCR System (Applied Biosystems). The primer sequences were as follows: β-actin, (forward primer: 5′-TCCTGTGGCATCCACGAAACT-3′; reverse primer: 5′-GAAGCATTTGCGGTGGACGAT-3′); E-cadherin, (forward primer: 5′-GTCACTGACACCAACGATAATCCT-3′; reverse primer: 5′-TTTCAGTGTGGT GATTACGACGTT-3′); vimentin (forward primer: 5′-TTGAACGCAAAGTGGAAT- 3′; reverse primer: 5′-AGGTCAGGCTTGGAAACA-3′); snail (forward primer: 5′-CAGATGAGGACAGTGGGAAAG-3′; reverse primer: 5′-CAGGCTGAGGTATTCCTTGTT-3′); slug (forward primer: 5′-CCCATTAGTGATGAAGAGGAAAGA-3′; reverse primer: 5′-CCAGGCTCACATATTCCTTGT-3′); twist1 (forward primer: 5′-AAGAAGTCTGCGGGCTGTG-3′; reverse primer: 5′-TCTGAATCTTGCTCAGCTTGT-3′); α-SMA (forward primer: 5′- GTGACTACTGCCGAGCGTG-3′; reverse primer: 5′- ATAGGTGGTTTCGTGGATGC-3′).

### Cell viability analysis

To access the cell survival, cells (5 × 10^3^ cells/well) were seeded in 96-well plates, and then, cells were incubated overnight in 95% air and 5% CO_2_ at 37 °C. After rLECT2 treatment for 48 h in serum-free medium, Alamar Blue reagent (10:1; Invitrogen) was added, and cells were incubated at 37 °C for 2∼4 h. Absorbance was measured with an ELISA reader (Dynex Technologies, Inc) at 570 to 620 nm. Cell viability was expressed as a percentage of absorbance in treated wells relative to that of untreated (control) wells.

### Colony formation assay

Liver cancer cells (3000 cells/well) were plated at 6-well plate, and cells were treated with rLECT2 in the medium containing 1% serum for 10 days. The colony (more than 50 cells) number was counted after fixing with 4 % paraformaldehyde (Sigma-Aldrich) and staining with crystal violet (Sigma-Aldrich).

### Cell invasion assay

The assay was performed as previously described (39). HCC cells were seeded in triplicate in the upper compartment of the chamber (1∼5 × 10^5^ cells in 50 *μ*l/well) and supplemented with serum-free medium containing rLECT2. The lower compartment was filled with 30 *μ*l of media containing 10% serum. Polycarbonate membrane with 8-*μ*m pore size (Nucleopore; Costar) was coated with Matrigel (BD Biosciences) to allow cell adhesion, and the upper and lower compartments were separated by the coated membrane. After incubation for 24 h in a humidified 5 % CO_2_ atmosphere chamber at 37 °C, cells on the upper side of the membrane were moved to the lower side. Migrated cells were fixed in absolute methanol and stained with 10% Giemsa solution (Sigma-Aldrich). Finally, the fixed cells were photographed by microscope with digital image system (Leica) and counted as mean ± SD. Every well was randomly selected five areas to quantify.

### Wound healing assay

The assay was performed as previously described (54). Hepatoma cells were seeded into 6-well plates (1 × 10^6^ cells/well) and grown to 90% confluence. A 100 μl pipette tip was used to scratch the cell monolayers. Then, the cells were washed with PBS and cultured in serum-free medium containing rLECT2 for an additional incubation. Images were taken at 0 h to 72 h with digital image system (Leica). The results were analyzed with ImageJ software (National Institutes of Health).

### Histological analysis

Immunohistochemical and immunofluorescence analysis were used as described previously (55). LECT2 (sc-47101, 2 μg/ml), CD133 (sc-30219, 2 μg/ml), CD44 (sc-9960, 2 μg/ml), β-catenin (sc-7199, 2 μg/ml), CD31 (sc-1506, 2 μg/ml), and HA-tag (sc-7392, 2 μg/ml) primary antibodies (Santa Cruz Biotechnology) were used in this study. TUNEL analysis was performed using the *in situ* Cell Death Detection Kit, Fluorescein (Roche), according to the manufacturer’s protocol.

### CD133^+^ and CD133^−^ cells sorting

The indirect magnetic-activated cell sorting (MACS) was used as described previously (56). In brief, N1-S1 cells were resuspended in ice-cold PBS and incubated with CD133 (MBS462020, 5 μg/ml) primary antibody (MyBioSource) for 30 min at 4 °C. After anti-CD133 removal, cells were incubated with goat-anti-rabbit IgG conjugated with MACS superparamagnetic microbeads (130–048–602) (Miltenyi Biotech GmbH, Bergisch-Gladbach, Germany) for 20 min at 4 °C. After wash by ice-cold PBS, CD133^+^ and CD133^−^ cells were separated using miniMACS magnetic cell separation column (Miltenyi Biotech GmbH).

### Sphere formation assay

The assay was performed as previously described (57). Liver cancer cells were suspended in serum-free Dulbecco's modified Eagle's medium/F12 (Gibco) medium containing B-27 (Gibco), 20 ng/ml EGF (PeproTech), and 20 ng/ml bFGF (PeproTech). HCC cells were plated at an ultralow-attachment 6-well plate (Corning Life Sciences), and HCC cells were treated with rLECT2. After 7 days, the spheres were observed and counted by optical microscope (Leica).

### SPCs assay

The assay was performed as previously described (57). 5 × 10^5^ HCC cells were plated at 6-well, and then cells were treated with rLECT2 in serum-free medium for 24 h. After cell harvesting, HCC cells were suspended in Hanks' Balanced Saline Solution (Gibco) containing with 5% fetal bovine serum (HyClone) and 5 μg/ml Hoechest 33342 (Sigma-Aldrich) for 90 min at 37 °C. In some cases, cells were incubated with Hoechst 33342 in the presence of 50 μM verapamil (Sigma-Aldrich) for reliable gating of SPCs. Subsequently, cells were centrifuged and resuspended in cold PBS containing 1 μg/ml propidium iodide (Sigma-Aldrich). SPCs were analyzed by flow cytometer (Beckman Coulter, Inc). The Hoechst 33342 was excited with the UV laser at 351 to 364 nm, and fluorescence was measured using a 515-nm SP filter (Hoechst blue) and a 608 EFLP optical filter (Hoechst red). A 540 DSP filter was used to separate the emission wavelengths.

### Counting of CD44^+^/CD133^+^ hCSCs by flow cytometry

After rLECT2 treatment for 24 h, HCC cells were incubated with CD44 (sc-9960, 5 μg/ml) (Santa Cruz Biotechnology) and CD133 (MBS462020, 5 μg/ml) (MyBioSource) primary antibodies for 30 min at room temperature and then washed 3 times with PBS. Next, signals were detected using an Alexa Fluor 488- or 546-conjugated IgG (Molecular Probes). Then, we used flow cytometer (Beckman Coulter, Inc) to determine the ratio of CD44+/CD133+ hCSCs. All the data were collected and analyzed using the *FlowJo* software (Tree Star).

### Rat aortic ring assay

The assay was performed as previously described (58). The thoracic aorta was dissected from euthanatized 8-week-old SD rats and followed by transverse section into the ring shape. The aorta rings were embedded in the 1 ml mixtures of Matrigel and MCDB131 media (Life technologies Ltd.) (1:1, *v*/*v*). To investigate the effect of LECT2 on microvessel sprouting, the aorta rings were incubated with rLECT2-containing medium for 7 days. The length of microvessel sprouts was measured with the microscope and digital image system (Leica). Five fields in each aortic ring were randomly selected for quantification.

### Statistical analysis

All between-group comparisons were analyzed by one-way ANOVA or a two-tailed student’s *t* test. The results are presented as mean ± SD, and *p* < 0.05 was considered statistically significant. We used GraphPad Prism 7.0 (GraphPad Software) for the statistical calculations. The quantification of histological data was performed by ImageJ (NIH). The survival rate in LECT2^High^ and LECT2^Low^ HCC patients and LECT2 expression in nontumor and tumor region of HCC patients from TCGA HCC dataset were analyzed by Kaplan–Meier Plotter (http://kmplot.com/analysis) and UCSC Xena (http://xena.ucsc.edu/), respectively. The correlation between LECT2 and target genes and the LECT2 expression in the tumor tissues of HCC patients with or without vascular invasion from TCGA cohort were also analyzed by UCSC Xena. The LECT2 level in human HCC from TCGA database with different histological grades and stages was analyzed by UALCAN (http://ualcan.path.uab.edu). The gene set enrichment analysis was analyzed by LinkedOmics database (http://www.linkedomics.org/admin.php).

## Data availability

All the data produced for this work are contained within the article and the supporting information.

## Supporting information

This article contains supporting information.

## Conflict of interest

The authors declare that they have no conflicts of interest with the contents of this article.
