# Supplementary material for: Leukocyte cell-derived chemotaxin 2 regulates epithelial-mesenchymal transition and cancer stemness in hepatocellular carcinoma
Source: J Biol Chem. 2022 Aug 31;298(10):102442. doi: 10.1016/j.jbc.2022.102442 (PMC9530851; doi:10.1016/j.jbc.2022.102442)
Supplement: Supporting Information [file mmc1.pdf]

Title: Leukocyte cell-derived chemotaxin 2 regulates epithelial-mesenchymal transition and cancer stemness in hepatocellular carcinoma

List of supporting information:

Figure S1: Exogenous LECT2 suppressed the expression of mesenchymal markers.

Figure S2: Ad-LECT2 infection repressed the cell mobility of Huh-7 cells.

Figure S3: LECT2 expression was associated with neovascularization of HCC based on LinkedOmics database.

Figure S4: Reduced LECT2 expression was shown in human HCC with vascular invasion.

Figure S5: LECT2 repressed angiogenic functions in endothelial cells and VEGF production in hepatoma cells.

Figure S6: Exogenous LECT2 treatment reduced the expressions of CSCs genes in N1-S1 cells.

Figure S7: LECT2 expression was correlated with the gene cluster of Wnt signaling pathway in HCC based on LinkedOmics database.

Figure S8: β-catenin signaling modulated cancer stemness, EMT and VEGF expression in hepatoma cells.

Figure S9: The activities of c-MET and β-catenin signaling were not negatively correlated with the differentiation status of liver cancer cells.

Figure S10: c-MET inhibitor suppressed β-catenin expression, EMT, VEGF production and stemness in Huh-7 cells.

Figure S11: LECT2 inhibited c-MET and β-catenin signaling in both $CD133^-$ and $CD133^+$ subpopulations of Huh-7 cells.

Figure S1

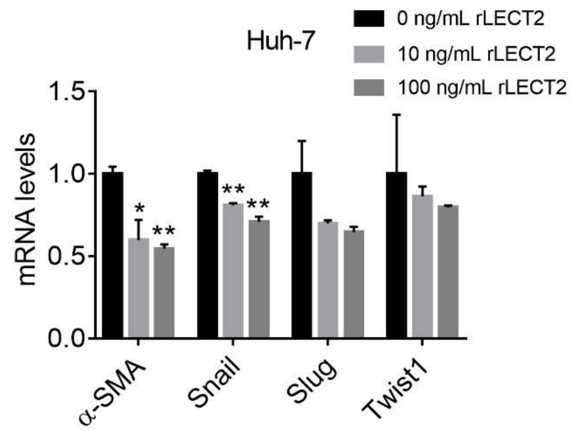

Figure S1. Exogenous LECT2 suppressed the expression of mesenchymal markers. qRT-PCR analysis for α-SMA, Snail, Slug and Twist1 in Huh7 cells after recombinant LECT2 treatment (10 and 100 ng/mL) for 24 h. Data were mean ± SD (*p < 0.05, **p < 0.01).

Figure S2

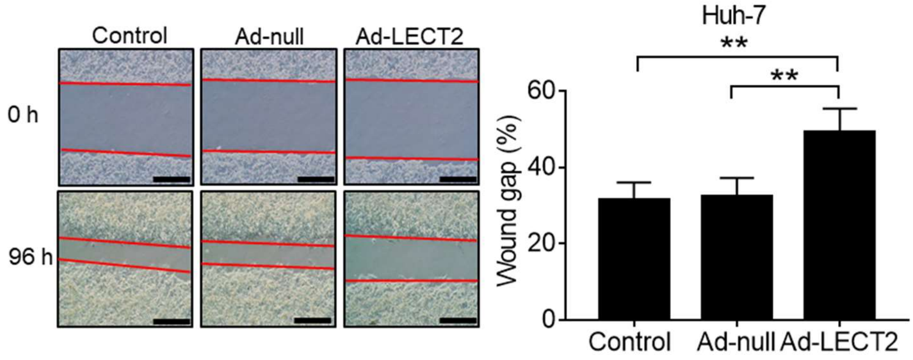

Figure S2. Ad-LECT2 infection repressed the cell mobility of Huh-7 cells. Scratch wound healing assay (from 0 to 52 h) for cell migration in Ad-LECT2- or Ad-null-infected (200 MOI for 48 h) Huh-7 cells. Scale bar = 200 μm. Data were mean ± SD (***p* < 0.01).

Figure S3

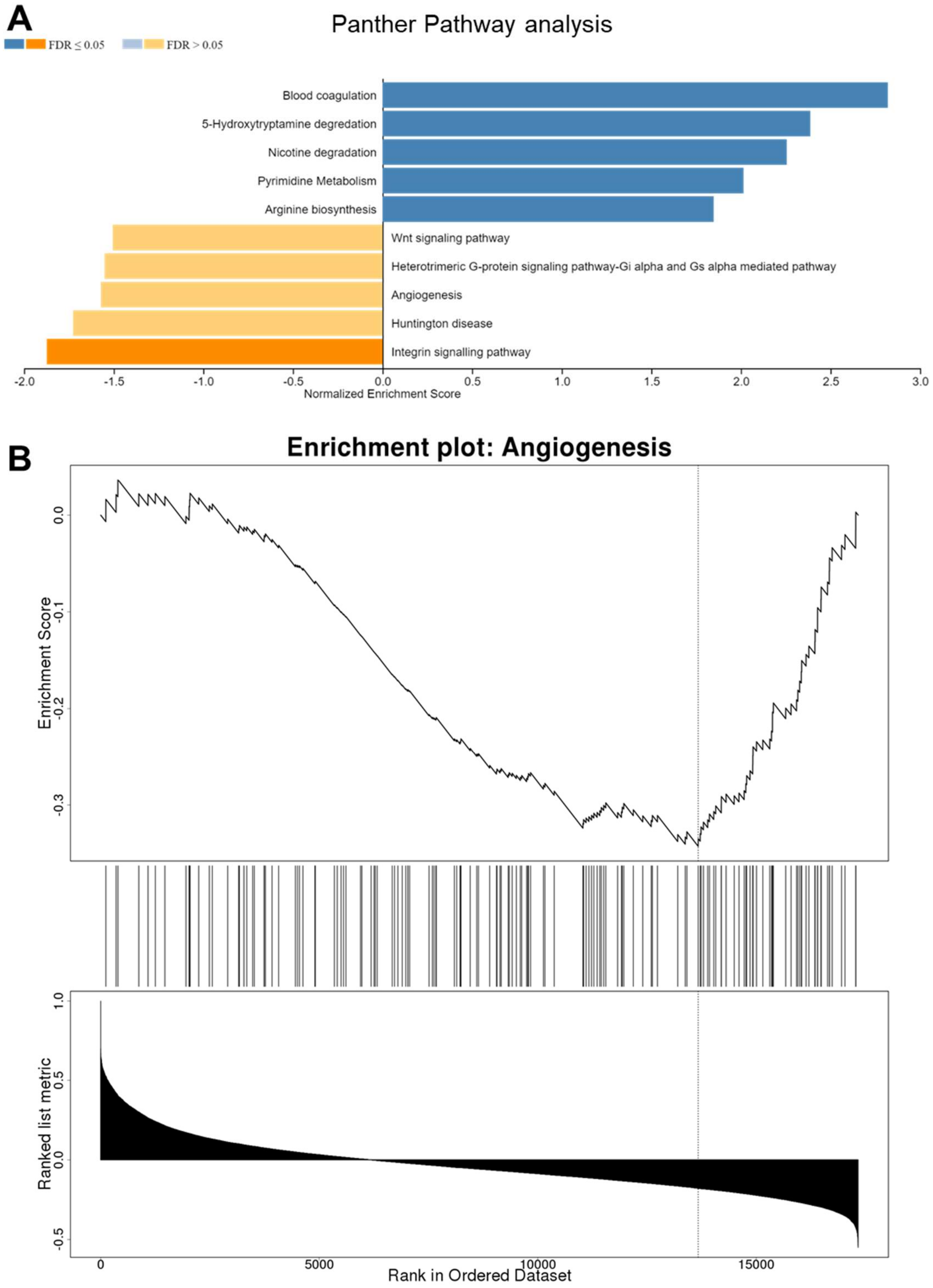

Figure S3. LECT2 expression was associated with neovascularization of HCC based on LinkedOmics database. (A) The Panther Pathway analysis for LECT2-related gene expressions of HCC. **(B)** The gene enrichment plot for the correlation of LECT2 expression and angiogenesis-related genes. False discovery rate (FDR) = 0.14343; ***p* = 0; normalized enrichment score = -1.5707.

Figure S4

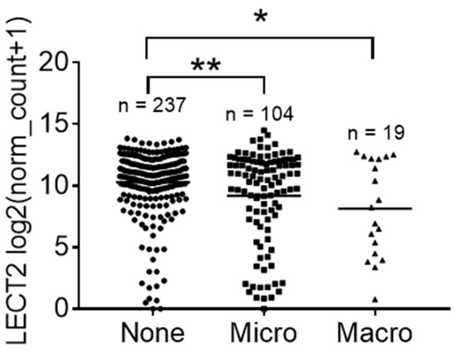

Figure S4. Reduced LECT2 expression was shown in human HCC with vascular invasion. TCGA analysis for LECT2 expression in human hepatoma tissues with or without vascular invasion. Micro = microvascular invasion, Macro = macrovascular invasion. Data were mean ± SD (*$p < 0.05$, **$p < 0.01$).

Figure S5

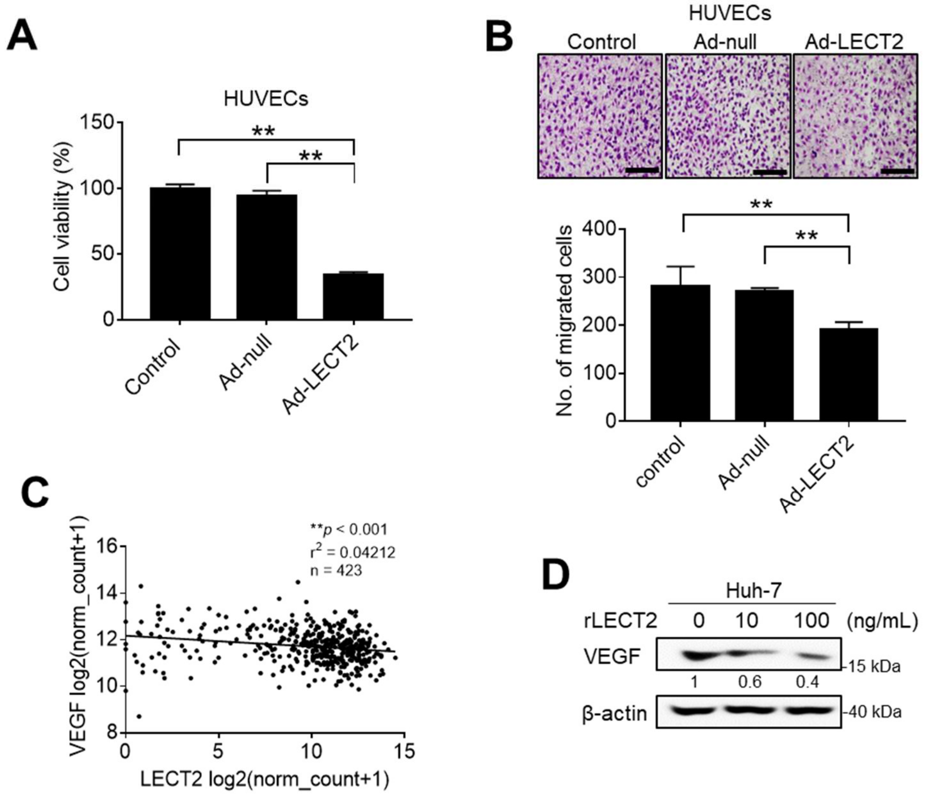

Figure S5. LECT2 repressed angiogenic functions in endothelial cells and VEGF production in hepatoma cells. (A) Alamar blue assay for cell viability in HUVECs after Ad-LECT2 or Ad-null infection (200 MOI) for 48 h. **(B)** Boyden chamber assay for cell invasion in Ad-LECT2- or Ad-null-infected (200 MOI for 48 h) HUVECs after seeding for 24 h. Scale bar = 200 μm. **(C)** TCGA analysis for the correlation between LECT2 and VEGF expressions in HCC patients. **(D)** Immunoblot analysis for VEGF in Huh-7 cells after rLECT2 (10 and 100 ng/mL) treatment for 24 h. Data were mean ± SD (**$p < 0.01$).

Figure S6

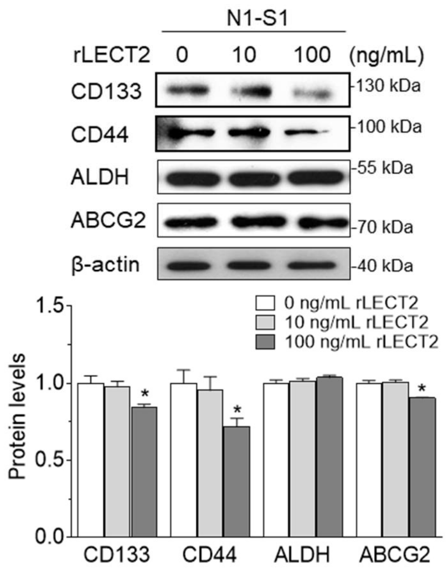

Figure S6. Exogenous LECT2 treatment reduced the expressions of CSCs genes in N1-S1 cells. Immunoblot analysis for CD133, CD44, ALDH and ABCG2 in N1-S1 cells after rLECT2 treatment for 24 h. The experiments of Figure S6 and Figure 7G were performed using the same sample and blot, and the identical β-actin bands are shown in these two panels. Data were mean ± SD (*$p < 0.05$).

Figure S7

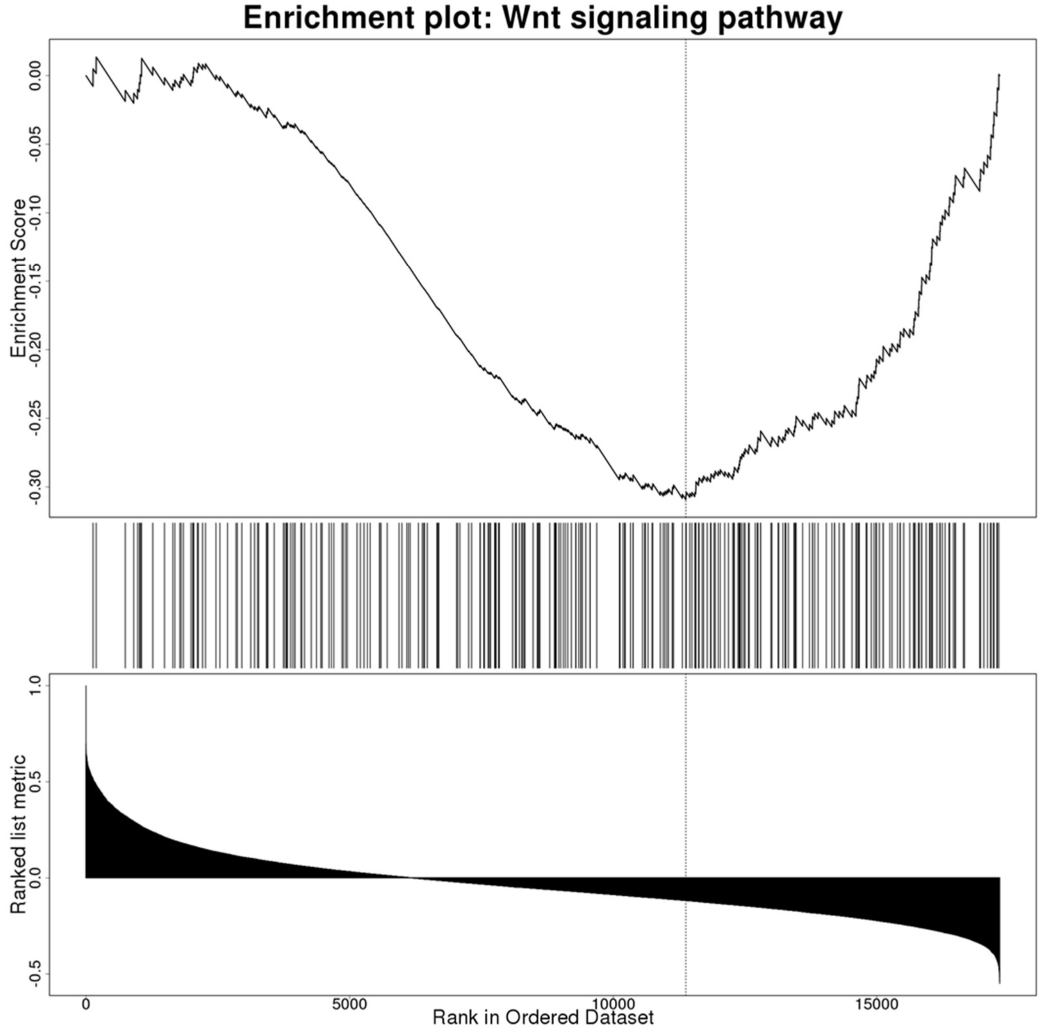

Figure S7. LECT2 expression was correlated with the gene cluster of Wnt signaling pathway in HCC based on LinkedOmics database. The gene enrichment plot for the correlation of LECT2 level and Wnt signaling pathway. False discovery rate (FDR) = 0.17576; **p = 0; normalized enrichment score = -1.5048.

Figure S8

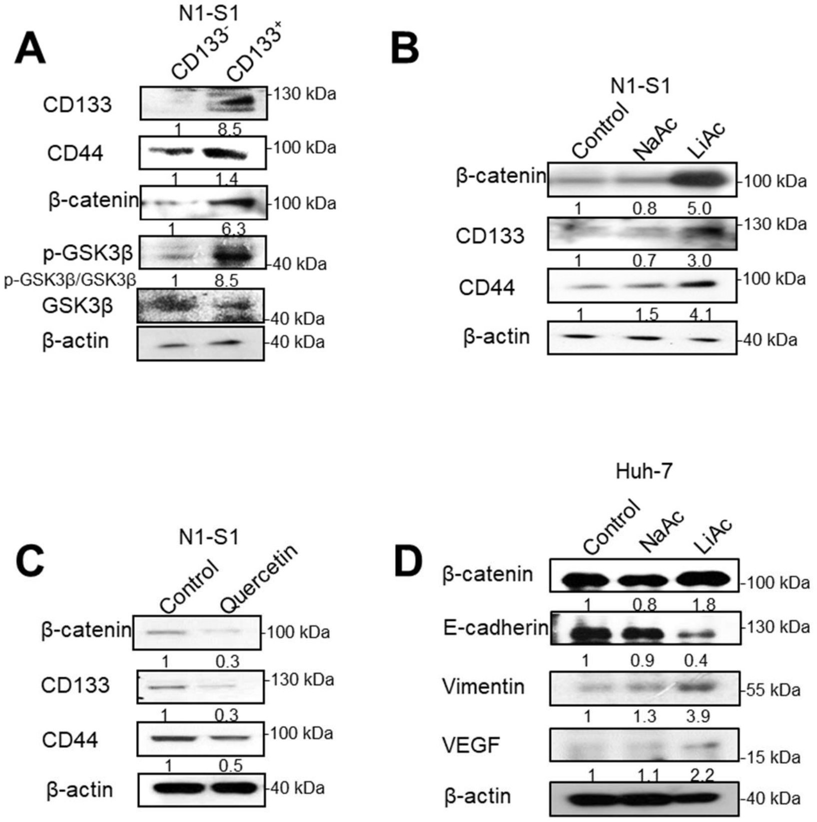

Figure S8. β-catenin signaling modulated cancer stemness, EMT and VEGF expression in hepatoma cells. (A) Immunoblot analysis for CD133, CD44, p-GSK3β, GSK3β and β-catenin in $CD133^-$ and $CD133^+$ N1-S1 cells. **(B)** Immunoblot analysis for β-catenin, CD44 and CD133 in N1-S1 cells after 20 mM NaAc (a vehicle control) or LiAc (GSK3β inhibitor) for 48 h. **(C)** Immunoblot analysis for β-catenin, CD133 and CD44 in N1-S1 cells after 50 μM quercetin (β-catenin inhibitor) for 48 h. **(D)** Immunoblot analysis for β-catenin, E-cadherin, vimentin and VEGF in Huh-7 cells after 20 mM NaAc or LiAc for 48 h.

Figure S9

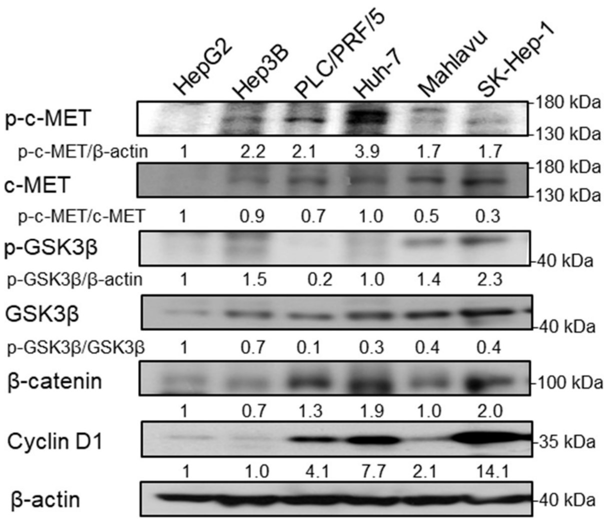

Figure S9. The activities of c-MET and β-catenin signaling were not negatively correlated with the differentiation status of liver cancer cells. Immunoblot analysis for p-c-MET, c-MET, p-GSK3β, GSK3β, β-catenin and Cyclin D1 in HepG2, Hep3B, PLC/PRF/5, Huh-7, Mahlavu and SK-Hep-1 liver cancer cells.

Figure S10

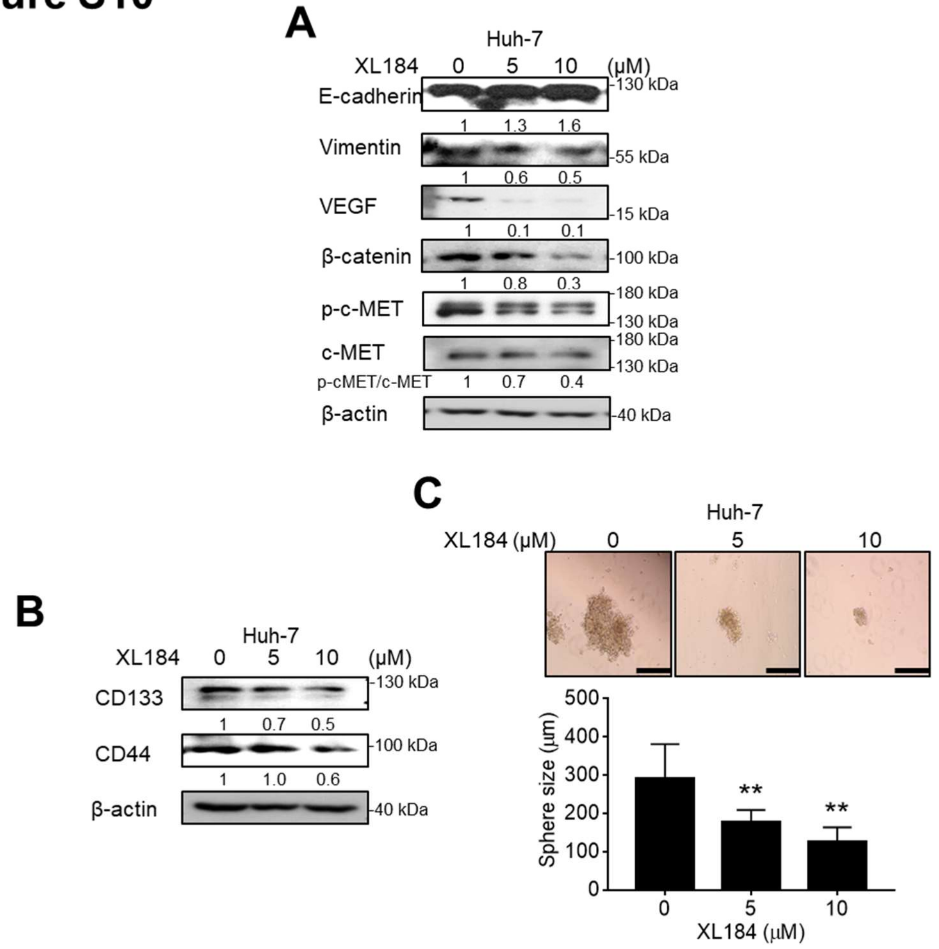

Figure S10. c-MET inhibitor suppressed β-catenin expression, EMT, VEGF production and stemness in Huh-7 cells. (A) Immunoblot analysis for E-cadherin, vimentin, β-catenin, p-c-MET, c-MET in Huh-7 cells after c-MET inhibitor XL184 treatment (5 and 10 μM) for 48 h. **(B)** Immunoblot analysis for CD133 and CD44 in Huh-7 cells after XL184 treatment (5 and 10 μM) for 48 h. **(C)** Tumor sphere assay for cell self-renewal in Huh-7 cells after XL184 treatment (5 and 10 μM) for 7 days. Sphere size were analyzed. Scale bar = 200 μm. Data were mean ± SD (***p* < 0.01).

Figure S11

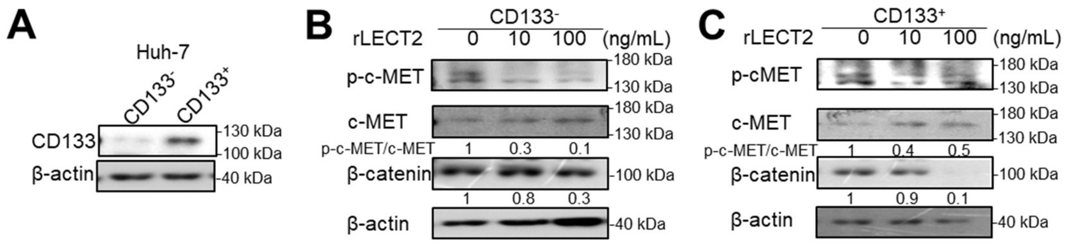

Figure S11. LECT2 inhibited c-MET and β-catenin signaling in both $CD133^-$ and $CD133^+$ subpopulations of Huh-7 cells. (A) Immunoblot analysis for CD133 in $CD133^-$ and $CD133^+$ Huh-7 cells. **(B)** Immunoblot analysis for p-c-MET, c-MET and β-catenin in $CD133^-$ Huh-7 cells. **(C)** Immunoblot analysis for p-c-MET, c-MET and β-catenin in $CD133^+$ Huh-7 cells.
